# A Novel Class of Cyclometalated Platinum(II) Complexes for Solution-Processable OLEDs

**DOI:** 10.3390/molecules27165171

**Published:** 2022-08-13

**Authors:** Dominique Roberto, Alessia Colombo, Claudia Dragonetti, Francesco Fagnani, Massimo Cocchi, Daniele Marinotto

**Affiliations:** 1 Department of Chemistry, University of Milan, UdR-INSTM, Via C. Golgi 19, 20133 Milan, Italy; 2 Istituto per la Sintesi Organica e la Fotoreattività (ISOF), Consiglio Nazionale delle Ricerche (CNR), Via P. Gobetti 101, 40129 Bologna, Italy; 3 Istituto di Scienze e Tecnologie Chimiche (SCITEC) “Giulio Natta”, Consiglio Nazionale delle Ricerche (CNR), Via C. Golgi 19, 20133 Milan, Italy

**Keywords:** coordination compounds, cyclometalated platinum(II) complexes, dipyridyl benzene ligand

## Abstract

Substitution of the chlorido ligand of cyclometalated [Pt (5-R-1,3-di(2-pyridyl) benzene)Cl] (R = methyl, mesityl, 2-thienyl, or 4-diphenylamino-phenyl) by 4-phenylthiazole-2-thiolate leads to related thiolato complexes, which were fully characterized. Their photophysical properties were determined in degassed dichloromethane solution. The emission color of the novel complexes can be easily tuned by the nature of the substituents on the terdentate ligand, as is the case for the parent chlorido complexes. Their luminescence Quantum Yield is high, with that of the compounds with the 2-thienyl or 4-diphenylamino-phenyl substituents being much higher than that of the related chloride complexes. The platinum complex with the cyclometalated 5-(2-thienyl)-1,3-di(2-pyridyl) benzene was used as the emitter for the fabrication of a yellow solution-processable OLED.

## 1. Introduction

**Figure 2:**
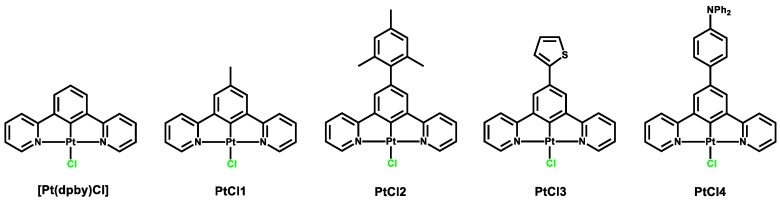
Structure of complexes **[Pt(dpby)Cl]** and **PtCl1–PtCl4**.

Transition metal complexes can find very useful applications in different fields because of the luminescence characteristics provided by the presence of the heavy metal atom. In fact, this allows for an efficient intersystem crossing which populates the excited triplet states, from which radiative emission can occur, even if theoretically forbidden. These appealing luminescence properties of transition metals, such as Platinum [1,2], can find application in different fields, from the production of light-emitting devices [3,4,5] to the use as dyes for bio-imaging and as biological probes [6,7,8]. In particular, this phenomenon can be observed in the case of cyclometalated Pt(II) complexes belonging to the family of **[Pt(dpyb)Cl]** compounds (dpyb = 1,3-di(2-piridyl) benzene, structure of the complex in Figure 2). The terdentate dpby chelating ligand offers a rigid environment around the platinum center, hampering non-radiative decays which could take place in the excited states. As a consequence, the absolute phosphorescence Quantum Yield of these complexes in deaerated solution reaches very high values (Φ_lum_ = 0.60 for the unsubstituted **[Pt(dpyb)Cl]** [9]).

The main fields in which the emission characteristics of this family of Pt(II) complexes have been tested until now, and can be furtherly exploited within, are the production of sensing devices and OLEDs [10,11,12,13,14,15,16], photodynamic therapy [17,18] and for bio-imaging [19,20,21,22,23]. Remarkably, the emission color of the compounds (and therefore also of the prepared devices) can be tuned by varying the substituents on the main scaffold of the terdentate ligand. Different substituents can be introduced on both the central benzene ring and the pyridines. Depending on the electron-donating or accepting properties of the substituents, the HOMO-LUMO gap of the complex can be modified and therefore the emission color can be tuned.

Thanks to the cyclometalating carbon atom in the *trans* position with respect to the ancillary Cl ligand in the square planar geometry of these compounds, the chloride can be easily replaced by other anionic species. Up to now, various ligands have been introduced in this way including isothiocyanates [11,22,23,24], azides [25], acetylides [26,27,28,29,30,31,32], isocyanides [33,34,35], phenolates [36] and thiolates [37,38,39,40,41]. While substitution with NCS or an acetylide maintains high Quantum Yields, the presence of a thiolate brings about different effects. A simple thioacetate [40] or differently substituted thiophenolates [37] result in a much lower Quantum Yield with respect to the parent chlorido compound, while 1-phenyl-1*H*-tetrazole-5-thiolate [41] provides a record value of 0.90.

Another possibility is to introduce substituents (such as 4-NPh_2_-phenyl [42]) on the pyridyl rings of the N^C^N ligands, leading to the expansion of the aromatic system and to higher QY (also in this case, up to 0.90).

**Figure 1:**
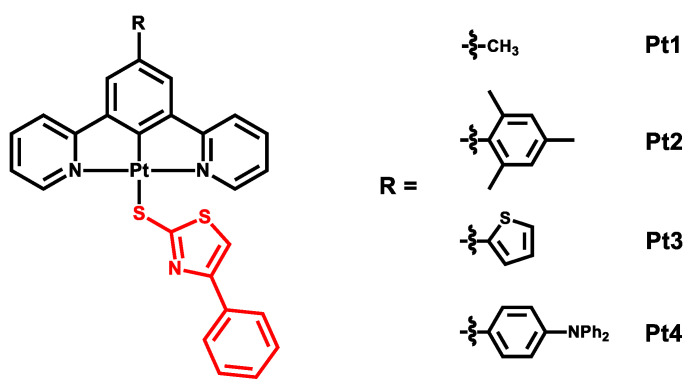
Structure of complexes **Pt1**–**Pt4**; in red, the new thiolate employed as ancillary ligand.

In this work, we presented four new complexes bearing a new sulfur-based ancillary ligand, namely a 4-phenylthiazole-2-thiolate. These complexes present different substituents on the benzene ring of the N^C^N ligand, i.e., a methyl, a mesityl, a 2-thienyl and a 4-diphenylamino-phenyl group (structure of the complexes in Figure 1).

All complexes were characterized from the absorption and luminescence point of view (Figures S7–S18), and compound **Pt3** was employed for the production of a yellow solution-processable OLED device.

## 2. Results and Discussion

Starting with the already known chlorido complexes **PtCl1**–**PtCl4** ([9,43], structures in Figure 2), four new compounds were synthesized with 4-phenylthiazole-2-thiolate as the ancillary ligand on the Pt(II) center (Figure 1). For all **Pt1**–**Pt4** complexes, UV-Vis absorption spectra were registered, together with emission, excitation, absolute Quantum Yield and lifetime measurements; the luminescence studies were carried out in deaerated dichloromethane solutions. Since the long-living triplet states of the platinum(II) complexes are efficiently quenched by molecular oxygen, three Freeze–Pump–Thaw (FPT) cycles were performed to remove the O_2_ present in the air and in the solution.

### 2.1. Photophysical Properties

**Figure 3:**
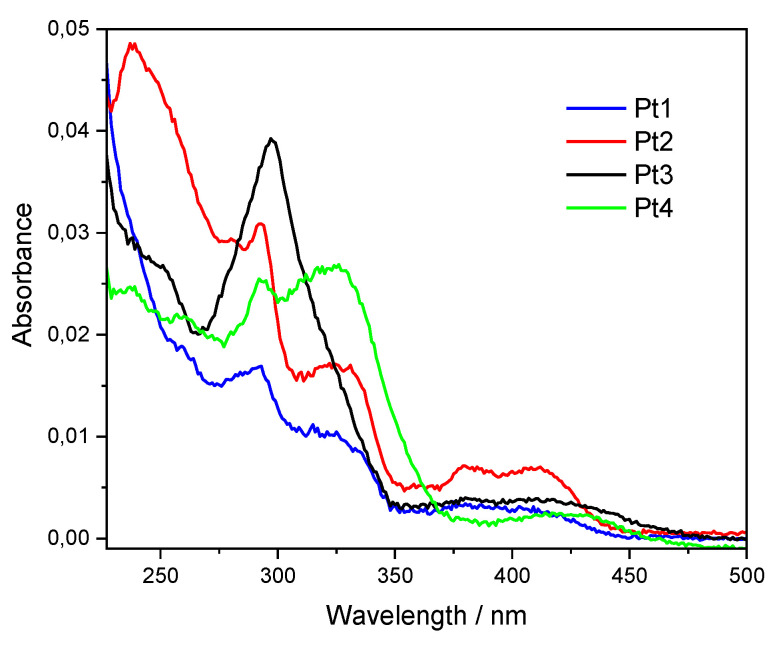
UV-Vis absorption spectra of **Pt1**–**Pt4** in dichloromethane, at a concentration of 1 × 10^−6^ M.

**Figure 4:**
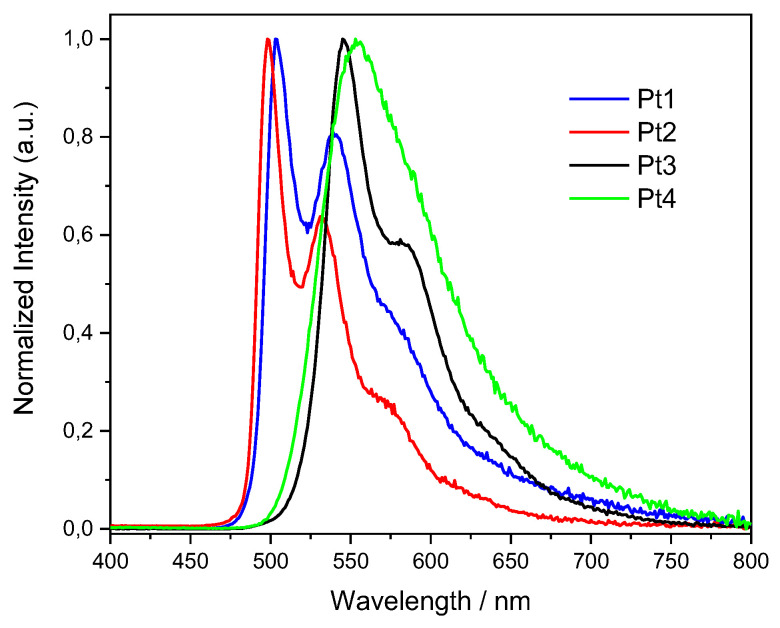
Emission spectra of **Pt1**–**Pt4** in dearated dichloromethane, at a concentration of 1 × 10^−6^ M.

Figure 3 shows the UV-Vis absorption spectra of the four new compounds **Pt1**–**Pt4**, while Figure 4 reports their normalized emission spectra; in both cases, the complexes were studied as dichloromethane solutions with a concentration of 1 × 10^−6^ M. The absorption spectra in CH_2_Cl_2_ at different concentrations were registered in order to calculate the molar extinction coefficients (ε); the spectra and a table with ε values are reported in the Supplementary Information. For all complexes, no aggregation was observed neither in absorption nor in the emission measurements.

The emission wavelengths, a comparison of the Quantum Yields before and after the FPT cycles, and the lifetimes are reported in Table 1, together with the values corresponding to the parent chlorido complexes **PtCl1**–**PtCl4**. Complete data and spectra are reported in the Supplementary Information.

It can be observed that the substitution of chloride with the 4-phenylthiazole-2-thiolate ligand exerts a very limited effect on the emission wavelength of the complexes, since a difference of only a few nm is present. Thus, the spectral region in which emission occurs is mainly determined by the substituent on the terdentate ligand. Nevertheless, by looking at **Pt3** and **Pt4**, an important effect on the absolute Φ_lum_ can be noticed upon the substitution of chloride with thiolate. Considering the reference values taken from literature [9,43], both the thienyl- and the 4-NPh_2_-phenyl-substituted complexes undergo a remarkable increase in Φ_lum_, from 54% to 89% and from 29% to 72%, respectively. Up to now, since very few studies exist in the field of NCN-Pt(II) complexes bearing thiolate ancillary ligands, it was not possible to point out a general trend in the QY values. Nevertheless, it can be noticed that the highest values among **Pt1**–**Pt4** were reached in the case of electron-rich substituents such as 2-thienyl and 4-NPh_2_-phenyl. As a future perspective, new thiolates and substituents could be tested, together with a theoretical investigation of the photophysical properties of such complexes.

Considering the lifetimes, it can be pointed out that the presence of the thiazole-based ancillary ligand did not bring about a change in the values in the case of **Pt1** (7.9 µs vs. 7.8 µs), **Pt2** (7.7 µs vs. 7.9 µs) and **Pt3** (19.1 µs vs. 20.5 µs); instead, only for **Pt4** was a remarkable increase observed, from 9.0 µs to 13.6 µs.

### 2.2. OLED Device Produced with Pt3

Since the novel complexes bearing a 4-phenylthiazole-2-thiolate ligand (**Pt1**–**Pt4**) are characterized by a high solubility in chlorinated solvents, they represent good candidates for application in the production of solution-processable devices, obtaining thin films of the compound by means of the spin-coating technique. Therefore, **Pt3**, i.e., the complex showing the highest value of Quantum Yield in the new class of dyes, was employed for the production of a solution-processable OLED device.

**Figure 5:**
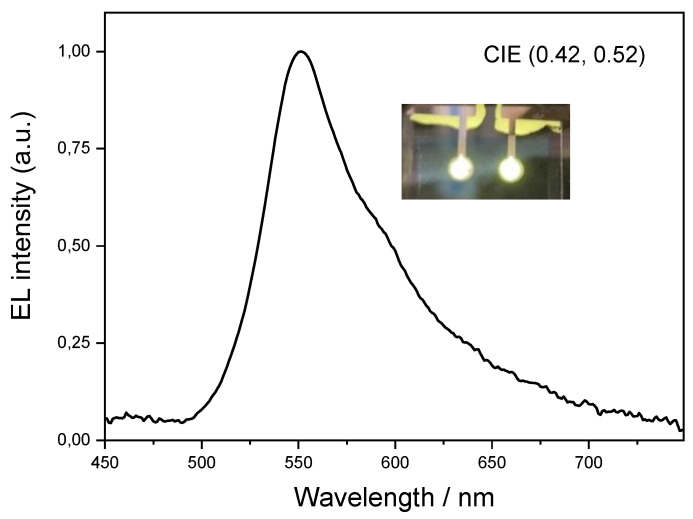
Electroluminescence spectra at 15 V of OLEDs based on **Pt3**. In the inset there is the photo of the resulting yellow OLED.

The EL spectrum of the OLED is shown in Figure 5. The OLED emission is in the yellow region, with CIE coordinates of (0.42, 0.52). The EL spectrum closely matches the emission of **Pt3** at a concentration of 1 × 10^−6^ M in dichloromethane (Figure 5). There is no significant contribution to the EL emission bands from the TBPi electron-transporting (hole-blocking) or TCTA binder layers, which is in agreement with a good charge carrier confinement within the EML and complete energy transfer from the excited states of TCTA (formed by charge carrier recombination) to the Pt complex.

**Figure 6:**
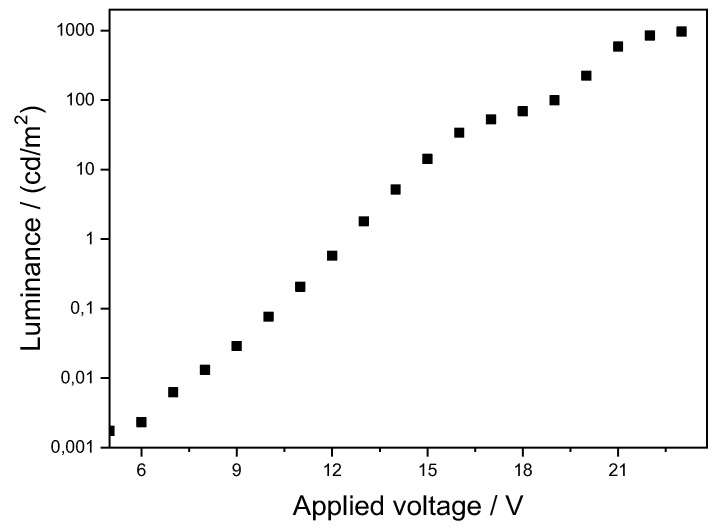
Luminance vs. applied voltage for the OLED device produced with 8% **Pt3** in the emissive layer.

The luminance as a function of the applied voltage of the OLED is shown in Figure 6. It is worth pointing out that the OLED performance observed with **Pt3** as the emitter is much better than that reported for a solution-processed OLED built with an N^C^N Pt complex bearing a chloride ancillary ligand [24] and similar to that observed for an OLED based on an N^C^N Pt complex having a 1-phenyl-1*H*-tetrazole-5-thiolate ancillary ligand [41].

## 3. Materials and Methods

All reagents and solvents were purchased from Sigma-Aldrich (St. Louis, MO, USA) and were used without further purification. The deuterated solvents for NMR measurements were purchased from Eurisotop (Saint-Aubin, France).

**Figure 7:**
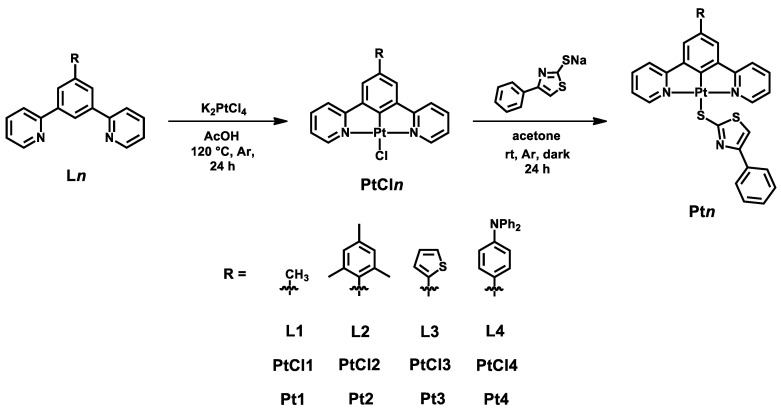
General synthesis of complexes **Pt1**–**Pt4**, starting from ligands **L1**–**L4**.

Ligands **L1**–**L4** (structure in Figure 7) were synthesized starting with 3,5-dibromotoluene (in the case of **L1**) or 1,3,5-tribromobenzene (for **L2**–**L4**) and by employing Pd-catalyzed Suzuki–Miyaura and/or Stille cross-coupling reactions to introduce the proper moieties on the N^C^N ligand.

In all cases, the known parent chlorido complexes **PtCl1**–**PtCl4** [9,43] were obtained by refluxing a mixture of the proper ligand (1 eq.) and K_2_PtCl_4_ (1.2 eq.) in glacial AcOH for 24 h under Argon atmosphere. The obtained precipitate was filtered, washed with H_2_O, MeOH and Et_2_O, and dried. Synthetic details and procedures are provided in the Supplementary Information.

Electronic absorption spectra were recorded at room temperature in CH_2_Cl_2_ solution, using a Shimadzu UV3600 spectrophotometer and quartz cuvettes with a 1 cm optical path length. Absolute photoluminescence Quantum Yields (Φ_lum_) were measured using a C11347 Quantaurus Hamamatsu Photonics K.K spectrometer. Steady-state and time-resolved fluorescence data were obtained using an FLS980 spectrofluorometer (Edinburg Instruments Ltd., Livingston, UK). A detailed description of the measurement techniques can be found in the Supplementary Information, together with the absorption and the luminescence spectra.

The device was built by using both dry and wet processes (sublimation in high vacuum and spin coating) in a pre-cleaned glass substrate made of indium tin oxide (ITO). Holes were injected from the ITO anode and passed through a 40 nm thick transporting layer made of PEDOT:PSS. Electrons were injected from an Al/LiF cathode and transported to the emitting layer (EML) by means of a layer of 2,2′,2′′-(1,3,5-benzinetriyl)-tris (1-phenyl-1*H*-benzimidazole) (TPBi, 30 nm thick). Charges recombined in the 40 nm thick EML made of a 4,4′,4′′-tris (N-carbazolyl-triphenylamine (TCTA) matrix, hosting **Pt3** (8% wt) as the emitter.

### General Synthesis of Complexes Pt1–Pt4

Compounds **Pt1**–**Pt4** were obtained (57–90% yields, see Supplementary Information) by stirring a mixture of the proper parent chloride complex (1 eq.) and of the sodium salt of 4-phenylthiazole-2-thiol (10 eq.) in acetone at room temperature in the dark under Argon atmosphere. After 24 h, the solution was evaporated to dryness under reduced pressure and dichloromethane was added to the solid residue in order to dissolve only the product. The sodium salt was filtered and the evaporation of the dichloromethane resulted in the desired product as an orange solid (Figure 2). All synthetic details and NMR spectra are reported in the Supplementary Information.

## 4. Conclusions

In conclusion, four novel cyclometalated Pt(II) complexes, bearing a variously substituted N^C^N 1,3-di(2-piridyl) benzene ligand and a 4-phenylthiazole-2-thiolate ancillary ligand, were easily prepared and well characterized. Their emission color can be easily tuned by the nature of the substituents on the terdentate ligand, as is the case for the parent chlorido complexes. However, their luminescence Quantum Yield can be much higher. Clearly, there is a need for the preparation and characterization of platinum(II) complexes with other sulfur co-ligands in order to understand the relationship between their nature and the emission properties of the compounds. In any case, the novel complexes with a 4-phenylthiazole-2-thiolate ligand reported here represent the first members of an interesting new class of soluble Pt(II) compounds which can be used as emitters for the fabrication of solution-processable OLEDs.

## Figures and Tables

**Table 1:** Key luminescence values for complexes **PtCl1**–**PtCl4** and **Pt1**–**Pt4**.

Complex	λ_max, em_/nm	Φ_lum before FPT_/%	Φ_lum after FPT_/%	τ/µs
**PtCl1** ^1^	505	-	68	7.8
**PtCl2** ^1^	501	-	62	7.9
**PtCl3** ^1^	548	-	54	20.5
**PtCl4** ^2^	557	-	29	9.0
**Pt1** ^3^	503	2.5	65	7.9
**Pt2** ^3^	498	3.5	55	7.7
**Pt3** ^3^	545	3.0	89	19.1
**Pt4** ^3^	554	2.5	72	13.6

^1^ From Ref. [9]. Luminescence Quantum Yields were determined by the method of continuous dilution, using quinine sulfate in 1 M H_2_SO_4_ as the standard; the estimated uncertainty is 20% or better. ^2^ From Ref. [43]. Luminescence Quantum Yield determined using [Ru(bpy)_3_]Cl_2_ as the standard. ^3^ This work; measured in dichloromethane solution (10^−6^ M) using a C11347 Quantaurus Hamamatsu Photonics K.K spectrometer.

## Data Availability

Data are contained within the article and Supplementary Material.

## Supplementary Materials

The following supporting information can be downloaded at: https://www.mdpi.com/article/10.3390/molecules27165171/s1, Figures S1–S6: synthetic pathways; Figures S7–S10: UV-Vis absorption spectra; Figures S11–S14: Emission and Excitation spectra; Figures S15–S18: lifetime measurements; Figures S19–S39: NMR spectra; Table S1: absorption maxima and ε values [44].

## Sample Availability

Samples of the compounds are not available.
