# Supplementary material for: A Novel Class of Cyclometalated Platinum(II) Complexes for Solution-Processable OLEDs
Source: Molecules. 2022 Aug 13;27(16):5171. doi: 10.3390/molecules27165171 (PMC9412278; doi:10.3390/molecules27165171)
Supplement: Supplementary file 1 [file molecules-27-05171-s001.zip › molecules-1857517-supplementary.pdf]

A Novel Class of Cyclometalated Platinum(II) Dyes for Solution-Processable OLEDs

Dominique Roberto [1], Alessia Colombo [1], Claudia Dragonetti [1], Francesco Fagnani [1]*, Massimo Cocchi [2] and Daniele Marinotto [3]

[1] Department of Chemistry, University of Milan, UdR-INSTM, Via C. Golgi 19, I-20133 Milan, Italy

[2] Istituto per la Sintesi Organica e la Fotoreattività (ISOF), Consiglio Nazionale delle Ricerche (CNR), via P. Gobetti 101, 40129 Bologna, Italy

[3] Istituto di Scienze e Tecnologie Chimiche (SCITEC) "Giulio Natta", Consiglio Nazionale delle Ricerche (CNR), via C. Golgi 19, 20133 Milan, Italy

* Correspondence: francesco.fagnani@unimi.it (F.F.); Tel.: +39-339-782-9874

General information 2
Synthesis of platinum complexes 4
Photoluminescence investigations of the complexes 13
NMR spectra 20

General information

Solvents were freshly distilled under argon from sodium / benzophenone (tetrahydrofuran, toluene) or from phosphorus pentoxide (dichloromethane). Anhydrous dimethylformamide was used as received for the supplier. Column chromatography purifications were performed with silica gel (Merck Geduran 60, 0.063–0.200 mm). NMR spectra were recorded on a Bruker AV III 300 MHz or AV III 400 MHz spectrometer. ^{1}H and ^{13}C chemical shifts are reported in parts per million (ppm) whilst the coupling constants (J) are expressed in Hz. The abbreviations reported in brackets refer to the multiplicities: s, d, t, q, m indicate respectively singlet, doublet, triplet, quartet, multiplet. Elemental analyses were carried out in the Department of Chemistry of the University of Milan.

Electronic absorption spectra of the three complexes in solution and solid state were obtained with a UV-3600i Plus UV-VIS-NIR Spectrophotometer (Shimadzu Italia S.r.l., Milan, Italy).

Luminescence measurements at room temperature were carried out in CH_2Cl_2 solution after three Freeze-Pump-Thaw cycles in order to remove dissolved oxygen.

Absolute photoluminescence quantum yield, Φ, was measured using a C11347 Quantaurus Hamamatsu Photonics K.K spectrometer. A description of the experimental setup and measurement method can be found in the article of K. Suzuki et al.[1] Φ was calculated through Equation:

$$\Phi = \frac{PN\ (Em)}{PN\ (Abs)} = \frac{\int \frac{\lambda}{hc}\left[I_{em}^{sample}(\lambda) - I_{em}^{reference}(\lambda)\right] d\lambda}{\int \frac{\lambda}{hc}\left[I_{exc}^{sample}(\lambda) - I_{exc}^{reference}(\lambda)\right] d\lambda}$$

where PN(Em) is the number of emitted photons, PN(Abs) the number of absorbed photons, λ the wavelength, h the Planck's constant, c the speed of light, I_{em}^{sample} and $I_{em}^{reference}$ the photoluminescence intensities of the sample solution and reference in CH_2Cl_2, I_{exc}^{sample} and $I_{exc}^{reference}$ the excitation light intensities of the sample solution and reference in CH_2Cl_2. PN(Em) is calculated in the wavelength interval [λ_i, λ_f], where λ_i is taken 10nm above the excitation wavelength, while λ_f is the upper end wavelength in the emission spectrum. The error made was estimated at around 5%.

Steady state and time-resolved fluorescence data were obtained using a FLS980 spectrofluorimeter (Edinburgh Instrument Ltd). Quartz cuvettes with 1 cm optical path length were used in a right-angle configuration between the excitation beam and the detection system. Emission spectra were

corrected for background intensity and quantum efficiency of the photomultiplier tube. Excitation spectra were corrected for the intensity fluctuation of a 450 W Xenon arc lamp.

Time-resolved fluorescence measurements were performed by either the time-correlated single photon counting technique with an Edinburgh Picosecond Pulsed Diode Laser (emitted wavelength 374 nm and 404 nm) or Multi-Channel Scaling technique with a microsecond pulsed Xenon flashlamp. In addition, time-resolved fluorescence curves were fitted using an exponential function:

$$I(\lambda, t) = \alpha(\lambda) \exp\left(\frac{-t}{\tau}\right)$$

where α (λ) is the amplitude at wavelength λ and τ is the lifetime. The quality of the fit was evaluated through the reduced χ^2 values.

Synthesis of platinum complexes

Synthesis of ligand L1

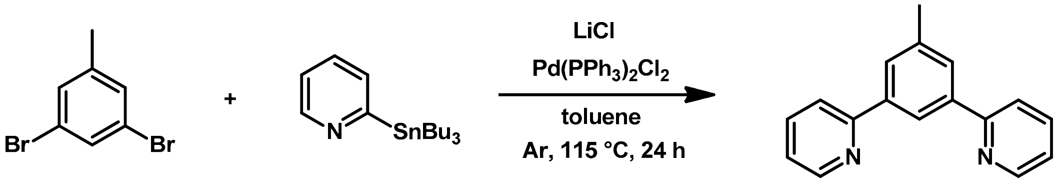

Figure S1. Synthesis of ligand **L1**.

Synthesis of L1. 3,5-dibromotoluene (200 mg), 2-tributylstannylpyridine (884 mg, 775 µL), LiCl (305 mg) and $Pd(PPh_3)Cl_2$ (28 mg) were added to dry toluene (5 mL) in a Schlenk tube and the mixture was stirred at reflux under Ar atmosphere. After 24 h a solution of NaOH (1 M, 10 mL), water and AcOEt were added and the phases were separated. The aqueous phase was extracted with AcOEt (3x), the organic phases were dried over Na_2SO_4 and evaporated.
The product was purified by flash chromatography on silica gel (eluent: hexane/AcOEt 7:3), obtaining 138 mg of **L1** (Yield = 70%).

^{1}H-NMR (400 MHz, $CDCl_3$) δ (ppm): 8.72 (2H, d, J = 4.6 Hz), 8.39 (1H, s), 7.91 (2H, s), 7.99-7-79 (6H, m), 7.25 (2H, dd, J = 4.6 Hz, J = 7.4 Hz), 2.53 (3H, s).

Synthesis of intermediates I2-I4

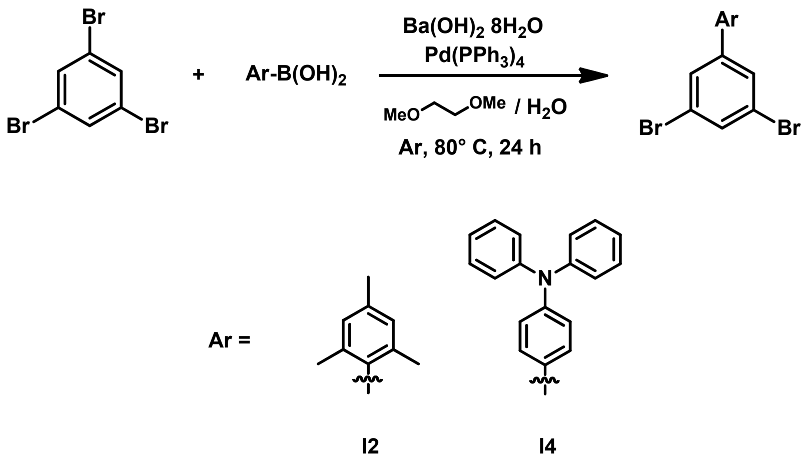

Figure S2. Synthesis of intermediates **I2** and **I4**.

Synthesis of I2. 1,3,5-tribromobenzene (200 mg), mesitylboronic acid (107 mg), $Ba(OH)_2 \cdot 8H_2O$ (301 mg) and $Pd(PPh_3)_4$ (22 mg) were added to a mixture of 1,2-dimethoxyethane (1.25 mL) and water (200 µL) in a Schlenk tube and the mixture was stirred at reflux under Ar atmosphere. After 24 h the reaction was cooled to rt, toluene and water were added and the phases were separated. The organic phase was washed with brine (2x) and water (2x), dried over Na_2SO_4 and evaporated at reduced pressure.

The product was purified by flash chromatography on silica gel (eluent: hexane), obtaining 174 mg of **I2** (Yield = 77%).

^{1}H-NMR (300 MHz, $CDCl_3$) δ (ppm): 7.66 (1H, t, J = 1.6 Hz), 7.26 (2H, d, J = 1.6 Hz), 6.94 (2H, s), 2.34 (3H, s), 2.02 (6H, s).

Synthesis of I4. 1,3,5-tribromobenzene (500 mg), 4-diphenylaminophenyl-boronic acid (383 mg), $Ba(OH)_2 \cdot 8H_2O$ (624 mg) and $Pd(PPh_3)_4$ (50 mg) were added to a mixture of 1,2-dimethoxyethane (3 mL) and water (500 µL) in a Schlenk tube and the mixture was stirred at reflux under Ar atmosphere. After 24 h the reaction was cooled to rt, toluene and water were added and the phases were separated. The organic phase was washed with brine (2x) and water (2x), dried over Na_2SO_4 and evaporated at reduced pressure.

The product was purified by flash chromatography on silica gel (eluent: hexane), obtaining 435 mg of **I4** (Yield = 69%).

^{1}H-NMR (400 MHz, $CDCl_3$) δ (ppm): 7.64 (2H, d, J = 1.7 Hz), 7.60 (1H, t, J = 1.7 Hz), 7.40 (2H, d, J = 8.7 Hz), 7.31 (4H, t, J = 8.2 Hz), 7.18-7.06 (8H, m).

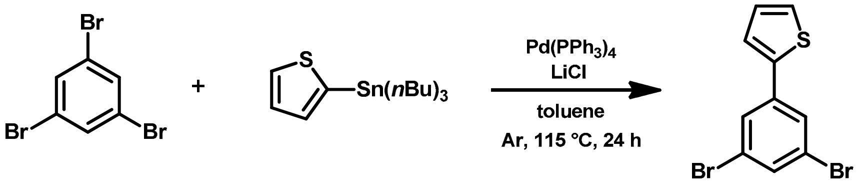

Figure S3. Synthesis of intermediate **I3**.

Synthesis of I3. 1,3,5-tribromobenzene (453 mg), 2-tributylstannylthiophene (587 mg, 502 μL), LiCl (304 mg) and $Pd(PPh_3)_2Cl_2$ (49 mg) were added to dry toluene (2 mL) in a Schlenk tube and the mixture was stirred at reflux under Ar atmosphere. After 24 the toluene was evaporated, water and DCM were added, and the phases were separated. The aqueous phase was extracted with DCM (2x), the organic phases were dried over Na_2SO_4 and evaporated.

The product was purified by flash chromatography on silica gel (eluent: hexane/AcOEt from 8:2 to 6:4), obtaining 224 mg of **I3** (Yield = 48%).

^{1}H-NMR (400 MHz, $CDCl_3$) δ (ppm): 7.69 (2H, s), 7.58 (1H, s), 7.37 (1H, d, J = 5.0 Hz), 7.33 (1H, d, J = 3.4 Hz), 7.11 (1H, dd, J = 3.4 Hz, J = 5.0 Hz).

Synthesis of ligands L2-L4

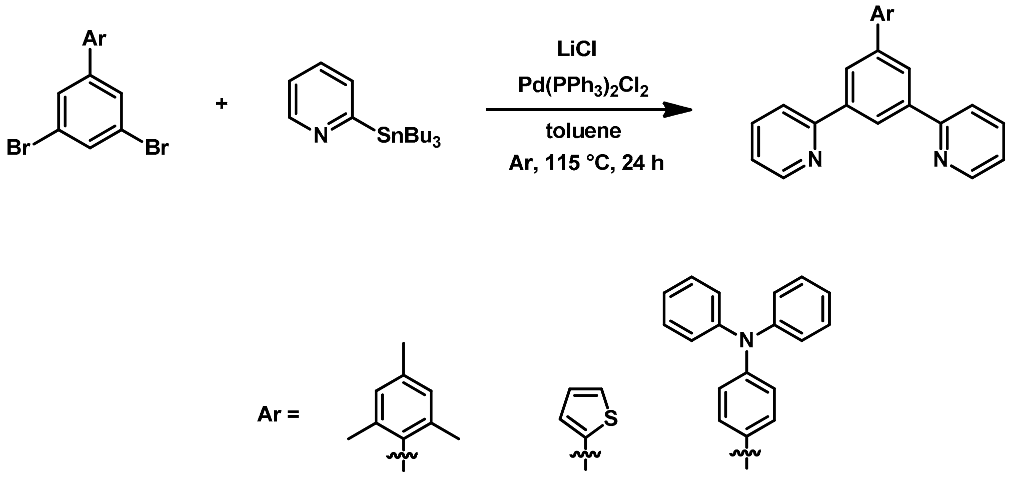

L2 L3 L4

Figure S4. Synthesis of ligands **L2-L4**.

Synthesis of L2. I2 (200 mg), 2-tributylstannylpyridine (624 mg, 550 µL), LiCl (213 mg) and $PdCl_2(PPh_3)_2$ (13 mg) were added to dry toluene (2 mL) in a Schlenk tube and the mixture was stirred at reflux under Ar atmosphere. After 24 h a solution of NaOH (1 M, 7 mL), water and AcOEt were added and the phases were separated. The aqueous phase was extracted with AcOEt (3x), the organic phases were dried over Na_2SO_4 and evaporated.

The product was purified by flash chromatography on silica gel (eluent: from hexane to hexane/AcOEt 9:1), obtaining 116 mg of **L2** (Yield = 59%).

^{1}H-NMR (400 MHz, $CDCl_3$) δ (ppm): 8.76-8.72 (2H, m), 8.70 (1H, t, J = 1.7 Hz), 7.89-7.84 (4H, m), 7.79 (2H, dd, J = 7.6 Hz, J = 8.0 Hz), 7.30-7.24 (2H, m, under residual $CHCl_3$), 6.99 (2H, s), 2.37 (3H, s), 2.11 (6H, s).

Synthesis of L3. I3 (667 mg), 2-tributylstannylpyridine (2.345 g, 2.0 mL), LiCl (802 mg) and $PdCl_2(PPh_3)_2$ (45 mg) were added to dry toluene (6.5 mL) in a Schlenk tube and the mixture was stirred at reflux under Ar atmosphere. After 24 h a solution of NaOH (1 M, 15 mL), water and AcOEt were added and the phases were separated. The aqueous phase was extracted with AcOEt (3x), the organic phases were dried over Na_2SO_4 and evaporated.

The product was purified by flash chromatography on silica gel (eluent: hexane/AcOEt 7:3), obtaining 489 mg of **L3** (Yield = 74%).

^{1}H-NMR (400 MHz, $CDCl_3$) δ (ppm): 8.76 (2H, d, J = 4.8 Hz), 8.54 (1H, t, J = 1.5 Hz), 8.33 (2H, d, J = 1.5 Hz), 7.89 (2H, d, J = 8.0 Hz), 7.80 (2H, dd, J= 7.6 Hz, J = 8.0 Hz), 7.53 (1H, d, J = 3.6 Hz), 7.34 (1H, d, J = 5.1 Hz), 7.28 (2H, dd, J = 4.8 Hz, J = 7.6 Hz), 7.14 (1H, dd, J = 3.6 Hz, J = 5.1 Hz).

Synthesis of L4. I4 (190 mg), 2-tributylstannylpyridine (456 mg, 400 μL), LiCl (151 mg) and $PdCl_2(PPh_3)_2$ (14 mg) were added to dry toluene (2.8 mL) in a Schlenk tube and the mixture was stirred at reflux under Ar atmosphere. After 24 h a solution of NaOH (1 M, 5 mL), water and AcOEt were added and the phases were separated. The aqueous phase was extracted with AcOEt (2x), the organic phases were dried over Na_2SO_4 and evaporated.
The product was purified by flash chromatography on silica gel (eluent: hexane/AcOEt 9:1), obtaining 118 mg of **L3** (Yield = 44%).

^{1}H-NMR (400 MHz, CD_2Cl_2) δ (ppm): 8.76 (dd, 2H, J = 4.8 Hz, J =), 8.67 (t, 1H, J = 1.6 Hz), 8.34 (d, 2H, J = 1.6 Hz), 7.96 (d, 2H, J = 7.8 Hz), 7.86 (2H, dd, J = 7.8 Hz, J = 8.0 Hz), 7.70 (d, 2H, J = 8.8 Hz), 7.36-7.30 (m, 6H), 7.24-7.17 (m, 6H), 7.09 (dd, 2H, J = 4.8 Hz, J = 8.0 Hz).

Synthesis of complexes PtCl1-PtCl4

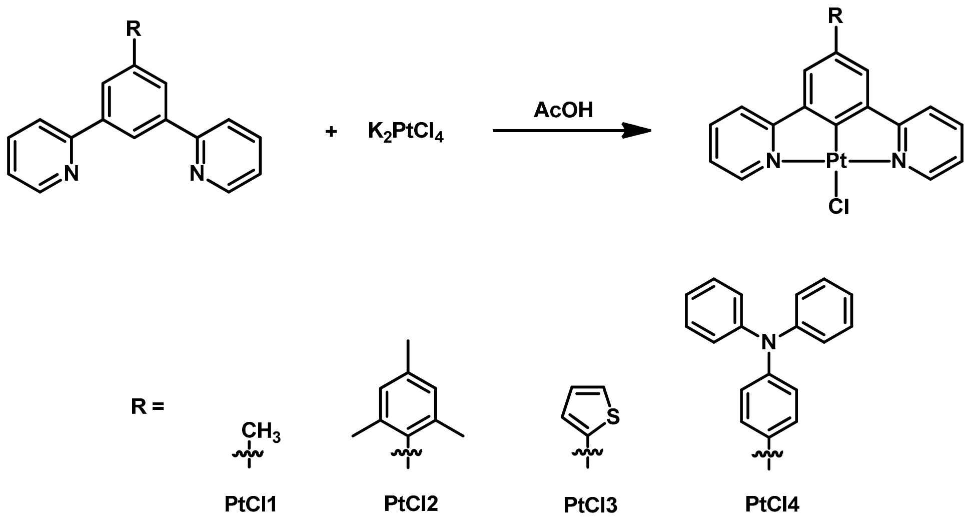

Figure S5. Synthesis of complexes **PtCl1-PtCl4**.

Synthesis of PtCl1. L1 (129 mg) and K_2PtCl_4 (260 mg) were added to glacial AcOH (8 mL) in a Schlenk tube and the mixture was stirred at reflux under Ar atmosphere. After 24 h some water was added to dissolve the excess K_2PtCl_4, the yellow precipitate was filtered on a Buchner funnel, washed with H_2O, MeOH and Et_2O, and dried, obtaining 229 mg of **PtCl1** (Yield = 92%).

^{1}H-NMR (400 MHz, CD_2Cl_2) δ (ppm): 9.28 (2H, d, J = 5.7 Hz, J(^{195}Pt) = 42 Hz), 8.00 (2H, dd, J = 7.7 Hz, J = 8.0 Hz), 7.73 (2H, d, J = 8.0 Hz), 7.37 (2H, s), 7.33 (2H, dd, J = 5.7 Hz, J = 7.7 Hz), 2.40 (3H, s).

Synthesis of PtCl2. L2 (60 mg) and K_2PtCl_4 (85 mg) were added to glacial AcOH (6 mL) in a Schlenk tube and the mixture was stirred at reflux under Ar atmosphere. After 24 h some water was added to dissolve the excess K_2PtCl_4, the orange precipitate was filtered on a Buchner funnel, washed with H_2O, MeOH and Et_2O, and dried, obtaining 45 mg of **PtCl2** (Yield = 45%).

^{1}H-NMR (400 MHz, CD_2Cl_2) δ (ppm): 9.34 (2H, d, J = 5.6 Hz, J(^{195}Pt) = 42 Hz), 8.00 (2H, dd, J = 7.6 Hz, J = 7.7 Hz), 7.72 (2H, d, J = 7.7 Hz), 7.37 (2H, dd, J = 5.6 Hz, J = 7.6 Hz), 2.37 (3H, s), 2.11 (6H, s).

Synthesis of PtCl3. L3 (120 mg) and K_2PtCl_4 (190 mg) were added to glacial AcOH (10 mL) in a Schlenk tube and the mixture was stirred at reflux under Ar atmosphere. After 24 h some water was added to dissolve the excess K_2PtCl_4, the orange precipitate was filtered on a Buchner funnel, washed with H_2O, MeOH and Et_2O, and dried, obtaining 140 mg of **PtCl3** (Yield = 67%).

^{1}H-NMR (400 MHz, CD_2Cl_2) δ (ppm): 9.33 (2H, d, J = 5.5, J(^{195}Pt) = 41 Hz), 8.06 (2H, dd, J = 7.8 Hz, J = 8.6 Hz), 7.85 (2H, d, J = 7.8 Hz), 7.78 (2H, s), 7.40 (1H, d, J = 3.5 Hz), 7.43-7.35 (3H, m), 7.17 (1H, dd, J = 3.5 Hz, J = 5.2 Hz).

Synthesis of PtCl4. L4 (80 mg) and K_2PtCl_4 (84 mg) were added to glacial AcOH (5 mL) in a Schlenk tube and the mixture was stirred at reflux under Ar atmosphere. After 24 h some water was added to dissolve the excess K_2PtCl_4, the orange-yellow precipitate was filtered on a Buchner funnel, washed with H_2O, MeOH and Et_2O, and dried, obtaining 91 mg of **PtCl4** (Yield = 77%).

^{1}H-NMR (300 MHz, CD_2Cl_2) δ (ppm): 9.35 (2H, d, J = 5.7 Hz, J(^{195}Pt) = 42 Hz), 8.05 (2H, dd, J = 7.6 Hz, J = 8.5 Hz), 7.85 (2H, d, J = 7.6 Hz), 7.75 (2H, s), 7.61 (2H, d, J = 8.6 Hz), 7.42-7.28 (6H, m), 7.24-7.14 (6H, m), 7.10 (2H, dd, J = 7.3 Hz, J = 8.0 Hz).

Synthesis of complexes Pt1-Pt4

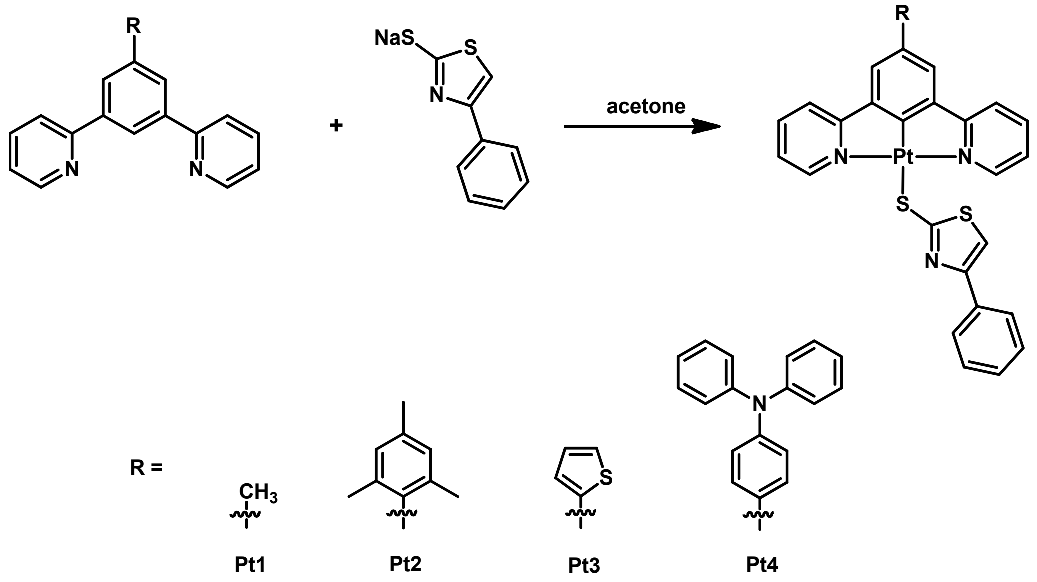

Figure S6. Synthesis of complexes **Pt1-Pt4**.

Synthesis of Pt1. PtCl1 (100 mg) and sodium 4-phenylthiazole-2-thiolate (316 mg) were added to acetone (100 mL) in a two-necked round-bottom flask and the mixture was stirred at rt in the dark under Ar atmosphere. After 24 h the acetone was evaporated at reduced pressure and DCM was added to dissolve the product; the evaporation of the DCM gave the product as an orange solid. Were obtained 80 mg of **PtCl1** (Yield = 57%).

^{1}H-NMR (400 MHz, CD_2Cl_2) δ (ppm): 9.41 (2H, d, J = 5.8 Hz, J(^{195}Pt) = 44 Hz), 7.99 (2H, dd, J = 7.8 Hz, J = 7.2 Hz), 7.87 (2H, d, J = 8.7 Hz), 7.76 (2H, d, J = 7.9 Hz), 7.44 (2H, s), 7.39 (2H, dd, J = 7.3 Hz, J = 8.0 Hz), 7.32-7.23 (3H, m), 6.98 (1H, s), 2.46 (3H, s).

^{13}C-NMR (100 MHz, CD_2Cl_2) δ (ppm): 153.51, 139.35, 128.41, 127.19, 125.91, 124.87, 123.64, 119.43, 109.84, 21.76.

Synthesis of Pt2. PtCl2 (50 mg) and sodium 4-phenylthiazole-2-thiolate (185 mg) were added to acetone (50 mL) in a two-necked round-bottom flask and the mixture was stirred at rt in the dark under Ar atmosphere. After 24 h the acetone was evaporated at reduced pressure and DCM was added to dissolve the product; the evaporation of the DCM gave the product as an orange solid. Were obtained 51 mg of **PtCl2** (Yield = 81%).

^{1}H-NMR (400 MHz, CD_2Cl_2) δ (ppm): 9.48 (2H, d, J = 5.6 Hz, J(^{195}Pt) = 44 Hz), 7.99 (2H, dd, J = 8.1 Hz, J = 8.2 Hz), 7.88 (2H, d, J = 7.5 Hz), 7.74 (2H, d, J = 7.6 Hz), 7.43-7.35 (4H, m), 7.34-7.26 (3H, m), 7.05-6.98 (3H, m), 2.38 (3H, s), 2.14 (6H, s).

Synthesis of Pt3. PtCl3 (30 mg) and sodium 4-phenylthiazole-2-thiolate (47 mg) were added to acetone (45 mL) in a two-necked round-bottom flask and the mixture was stirred at rt in the dark under Ar atmosphere. After 24 h the acetone was evaporated at reduced pressure and DCM was added to dissolve the product; the evaporation of the DCM gave the product as an orange solid.
Were obtained 35 mg of **PtCl3** (Yield = 90%).

^{1}H-NMR (400 MHz, CD_2Cl_2) δ (ppm): 9.41 (2H, d, J = 5.7 Hz, J(^{195}Pt) = 44 Hz), 8.02 (2H, dd, J = 7.7 Hz, J = 8.6 Hz), 7.90-7.80 (4H, m), 7.78 (2H, s), 7.47 (1H, d, J = 3.5 Hz), 7.42-7.36 (3H, m), 7.20-7.16 (3H, m), 6.99 (1H, s).

Synthesis of Pt4. PtCl4 (40 mg) and sodium 4-phenylthiazole-2-thiolate (172 mg) were added to acetone (60 mL) in a two-necked round-bottom flask and the mixture was stirred at rt in the dark under Ar atmosphere. After 24 h the acetone was evaporated at reduced pressure and DCM was added to dissolve the product; the evaporation of the DCM gave the product as an orange solid.
Were obtained 31 mg of **PtCl4** (Yield = 63%).

^{1}H-NMR (300 MHz, CD_2Cl_2) δ (ppm): 9.45 (2H, d, J = 5.7 Hz, J(^{195}Pt) = 41 Hz), 8.02 (2H, dd, J = 8.0 Hz, J = 8.4 Hz), 7.91-7.81 (4H, m), 7.79 (2H, s), 7.62 (2H, d, J = 8.6 Hz), 7.44-7.05 (15H, m), 6.99 (1H, s).

Photophysical characterization of the complexes

Here are reported all data and spectra related to the photophysical characterization of complexes **Pt1-Pt4**.

Figures S7-S10 report the UV-Vis absorption spectra at different concentrations, while the normalized Excitation and Emission spectra are shown in **Figures S11-S14**.

Table S1 lists the values of molar extinction coefficients for the absorption maxima, while **Figures S15-S18** show the decays measured to calculate the lifetimes.

Absorption spectra

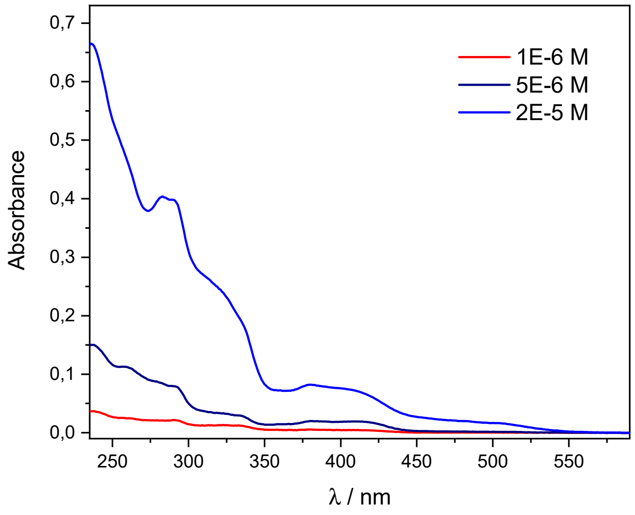

Figure S7. UV-Vis absorption spectra of complex **Pt1** in CH_2Cl_2 at different concentrations.

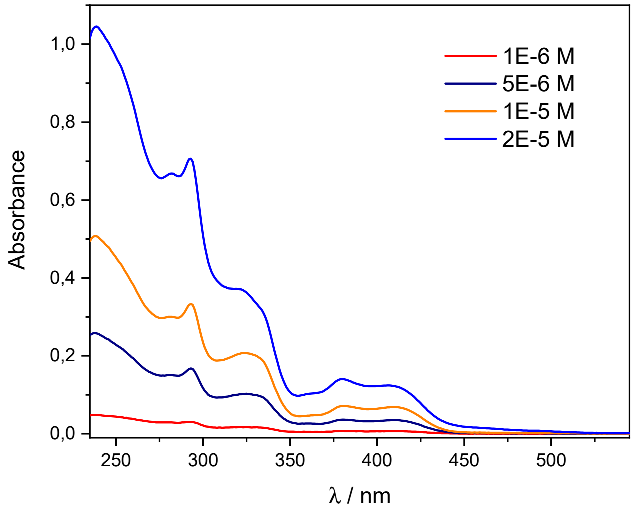

Figure S8. UV-Vis absorption spectra of complex **Pt2** in CH_2Cl_2 at different concentrations.

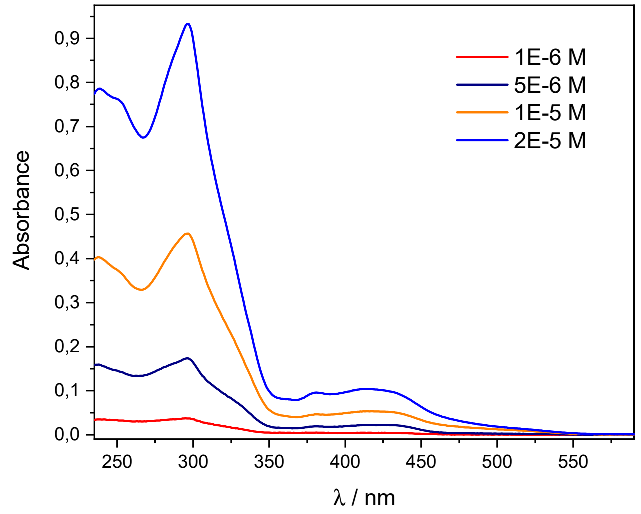

Figure S9. UV-Vis absorption spectra of complex **Pt3** in CH_2Cl_2 at different concentrations.

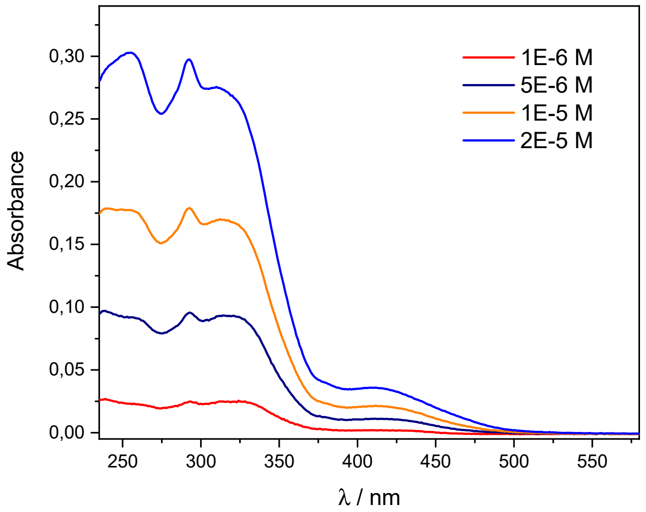

Figure S10. UV-Vis absorption spectra of complex **Pt4** in CH_2Cl_2 at different concentrations.

Molar extinction coefficients

Having measured the UV-Vis absorption spectra at different concentrations for all complexes **Pt1-Pt4**, it was possible to calculate the molar extinction coefficients (ε) for the absorption maxima of the compounds.

Table S1 lists the ε values for **Pt1-Pt4**.

Table S1. Absorption maxima and corresponding values of ε for complexes **Pt1-Pt4**.

**Complex**		$\lambda_{max, abs}$ / nm	ε / (10^3 cm^{-1} M^{-1})
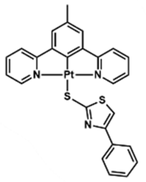	**Pt1**	236<br>283<br>380<br>410	33.4<br>20.4<br>4.1<br>3.5
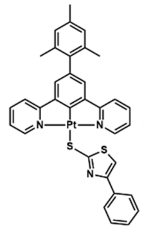	**Pt2**	238<br>293<br>380<br>408	52.4<br>35.5<br>7.0<br>6.1
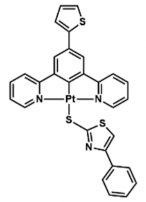	**Pt3**	238<br>297<br>381<br>415	40.2<br>48.4<br>4.9<br>5.3
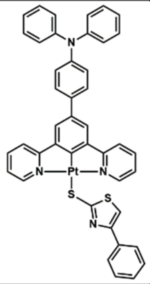	**Pt4**	253<br>292<br>408	14.6<br>14.2<br>1.7

Excitation and Emission spectra

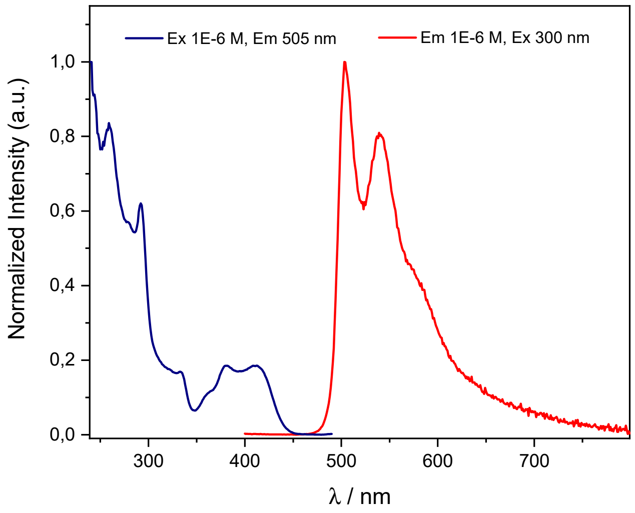

Figure S11. Normalized excitation and emission spectra of a $1 \cdot 10^{-6}$ M solution of complex **Pt1** in CH_2Cl_2, after three Freeze-Pump-Thaw cycles.

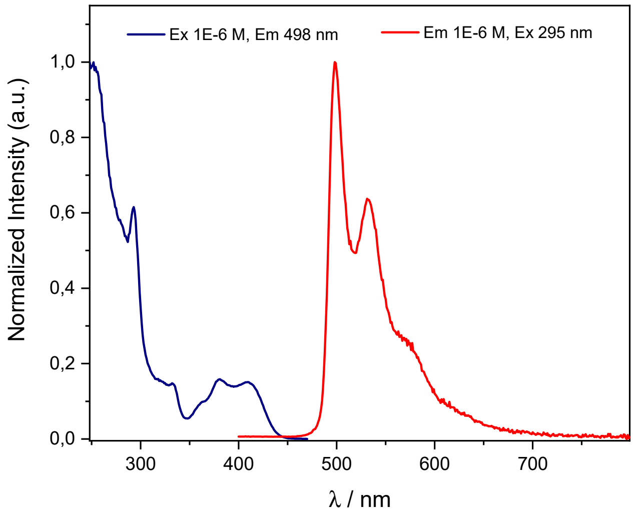

Figure S12. Normalized excitation and emission spectra of a $1 \cdot 10^{-6}$ M solution of complex **Pt2** in CH_2Cl_2, after three Freeze-Pump-Thaw cycles.

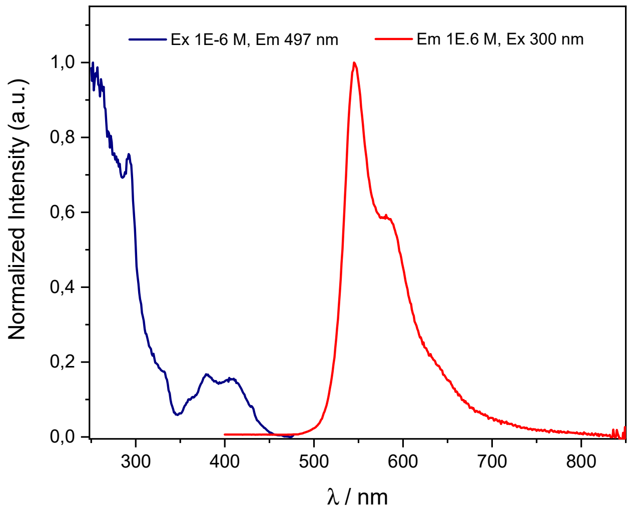

Figure S13. Normalized excitation and emission spectra of a 1·10^{-6} M solution of complex **Pt3** in CH_2Cl_2, after three Freeze-Pump-Thaw cycles.

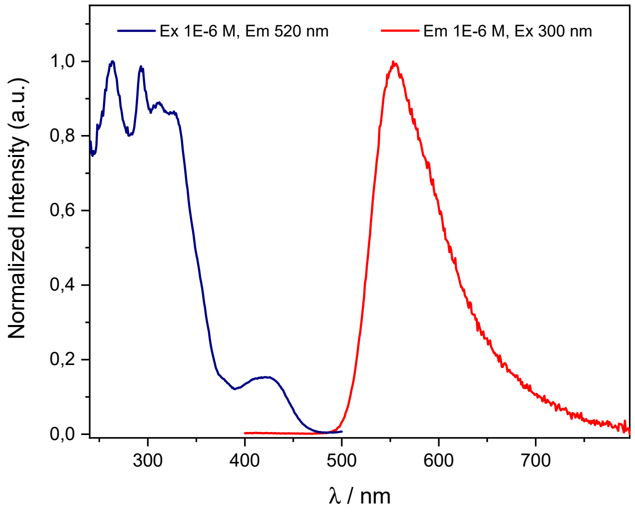

Figure S14. Normalized excitation and emission spectra of a 1·10^{-6} M solution of complex **Pt4** in CH_2Cl_2, after three Freeze-Pump-Thaw cycles.

Lifetimes

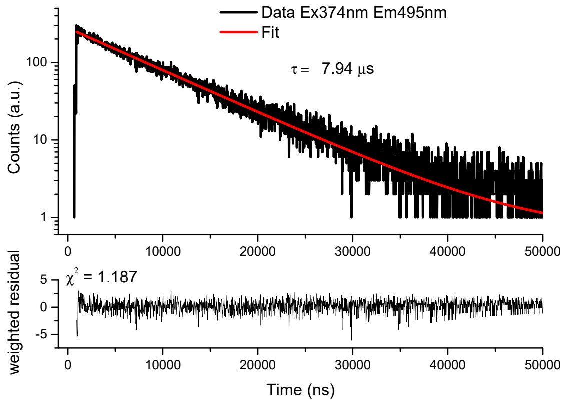

Figure S15. Lifetime measurements of a $1\cdot10^{-6}$ M dichloromethane solution of **Pt1**.

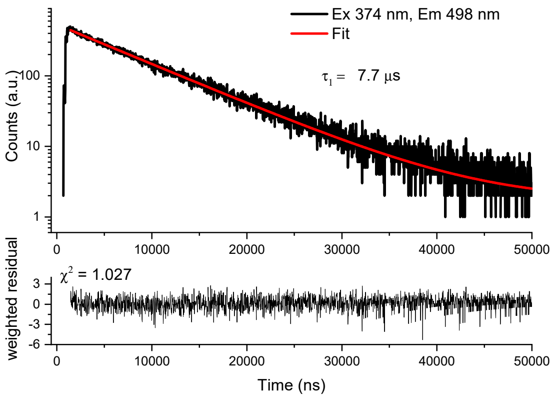

Figure S16. Lifetime measurements of a $1\cdot10^{-6}$ M dichloromethane solution of **Pt2**.

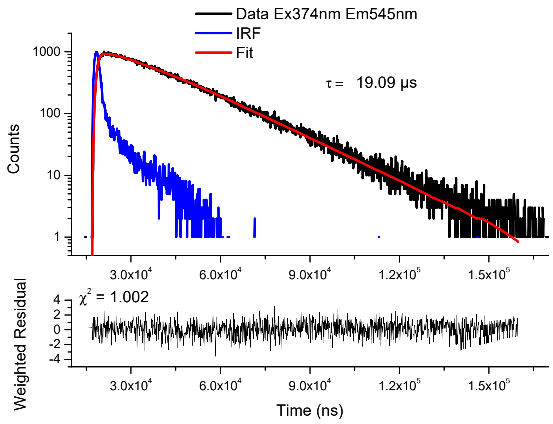

Figure S17. Lifetime measurements of a $1 \cdot 10^{-6}$ M dichloromethane solution of **Pt3**.

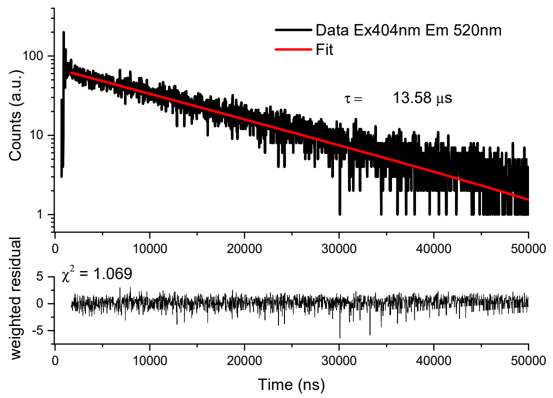

Figure S18. Lifetime measurements of a $1 \cdot 10^{-6}$ M dichloromethane solution of **Pt4**.

NMR spectra

L1

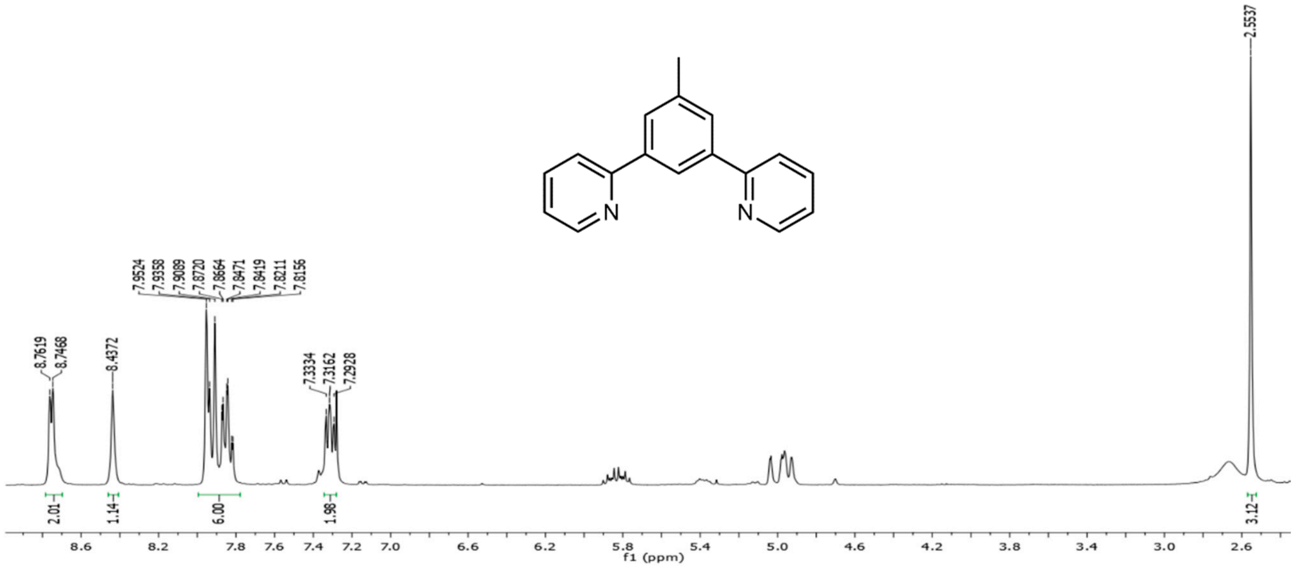

Figure S19. ^{1}H NMR of **L1**.

I2

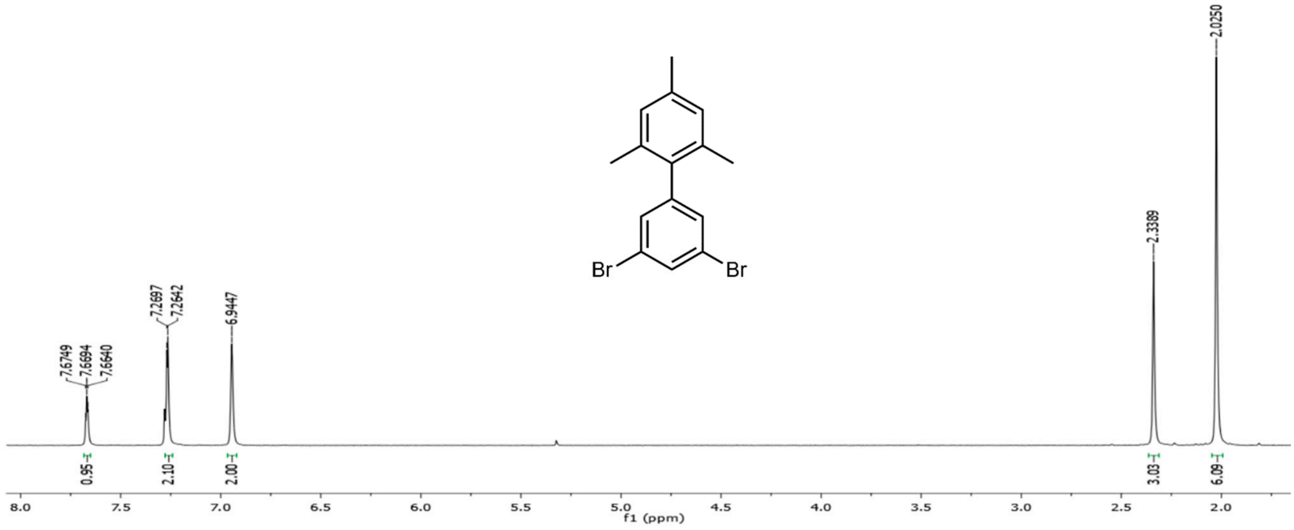

Figure S20. ^{1}H NMR of **I2**.

I3

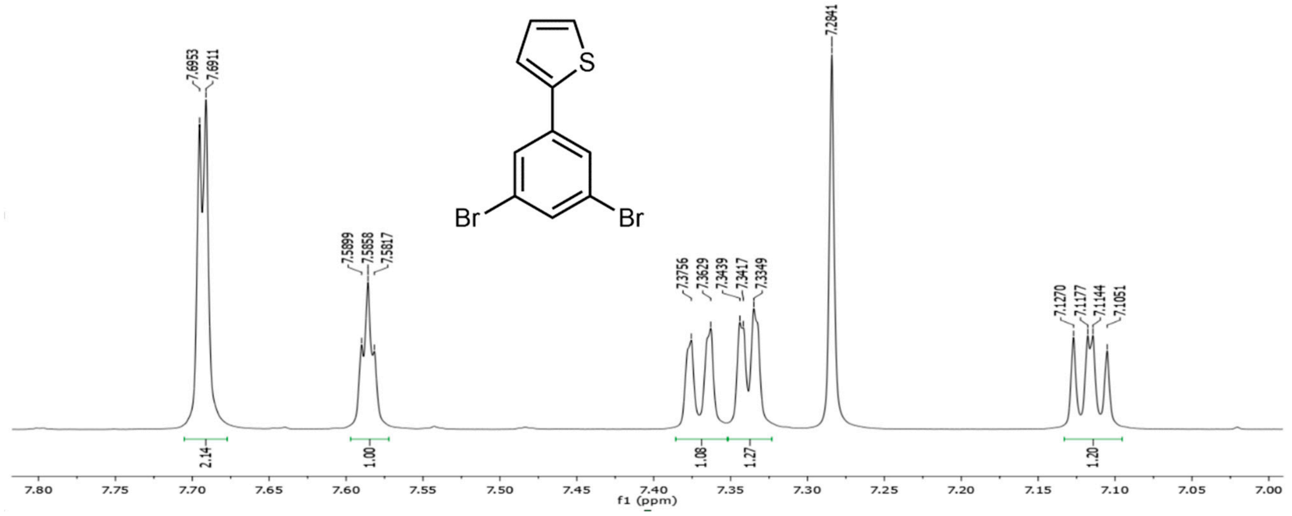

Figure S21. ^{1}H NMR of **I3**.

I4

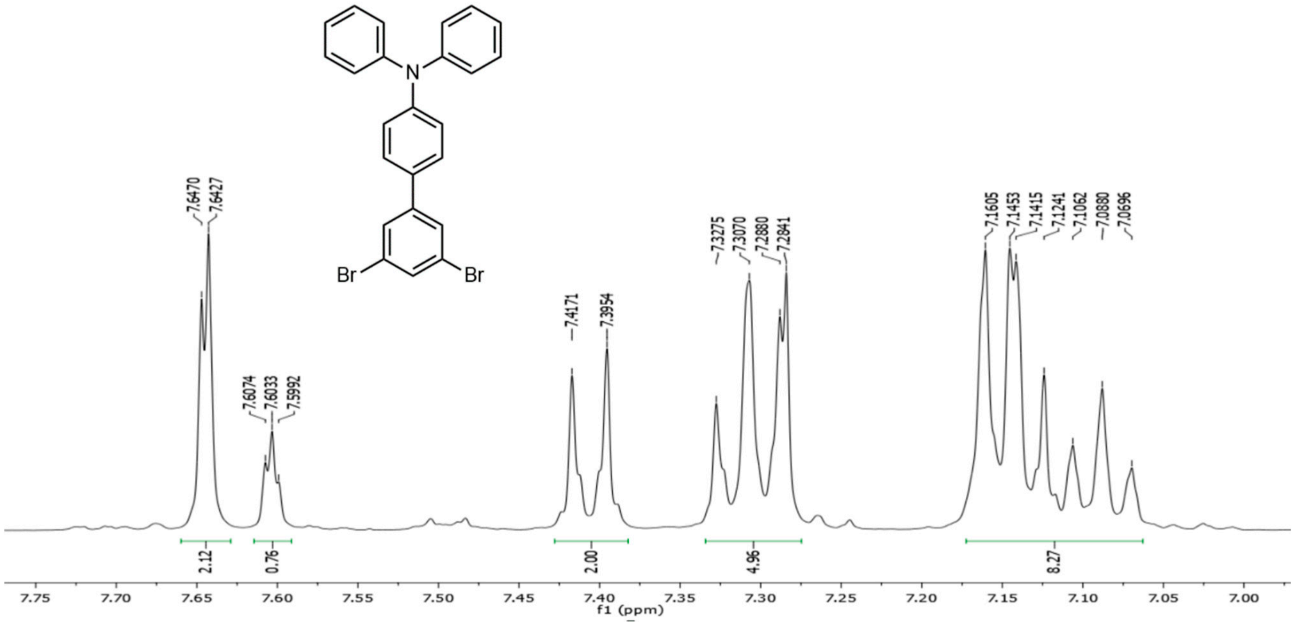

Figure S22. ^{1}H NMR of **I4**.

L2

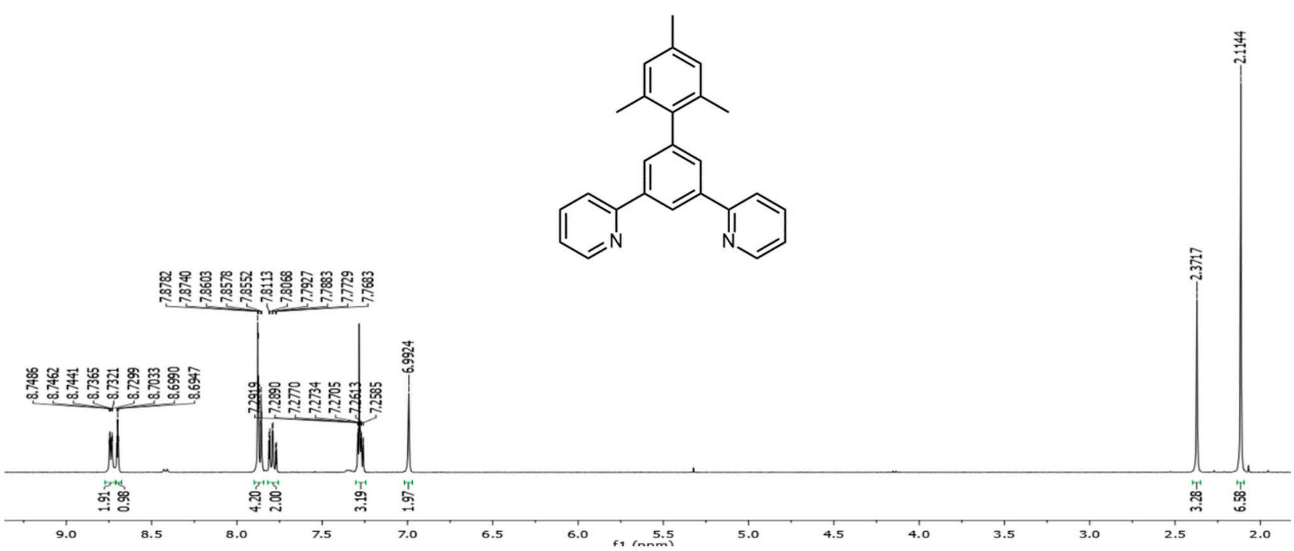

Figure S23. ^{1}H NMR of **L2**.

L2 – aromatic region

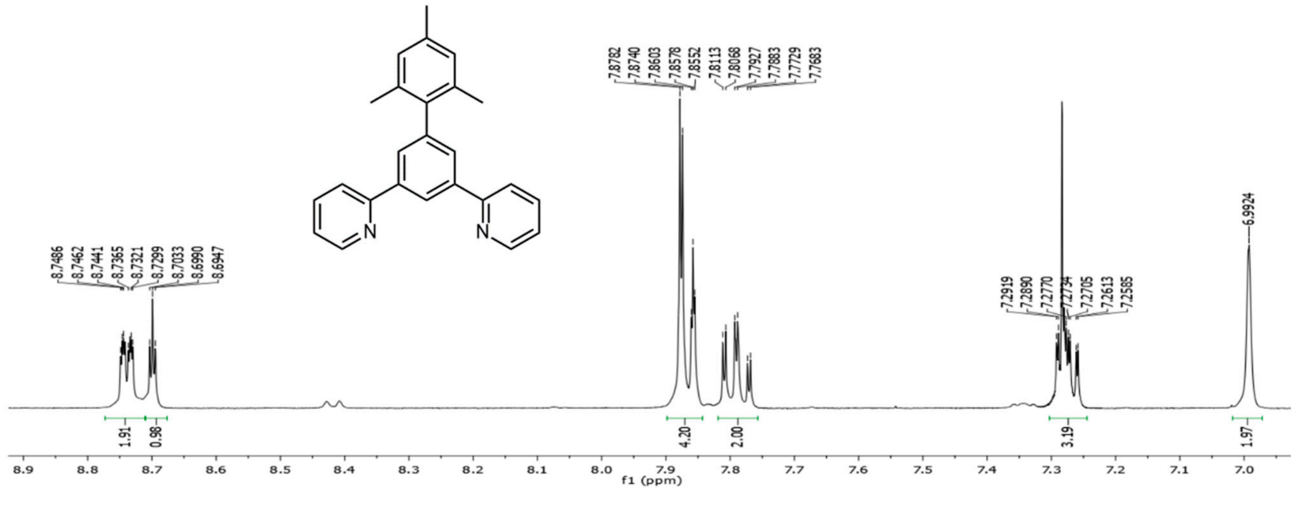

Figure S24. ^{1}H NMR of **L2**, aromatic region.

L3

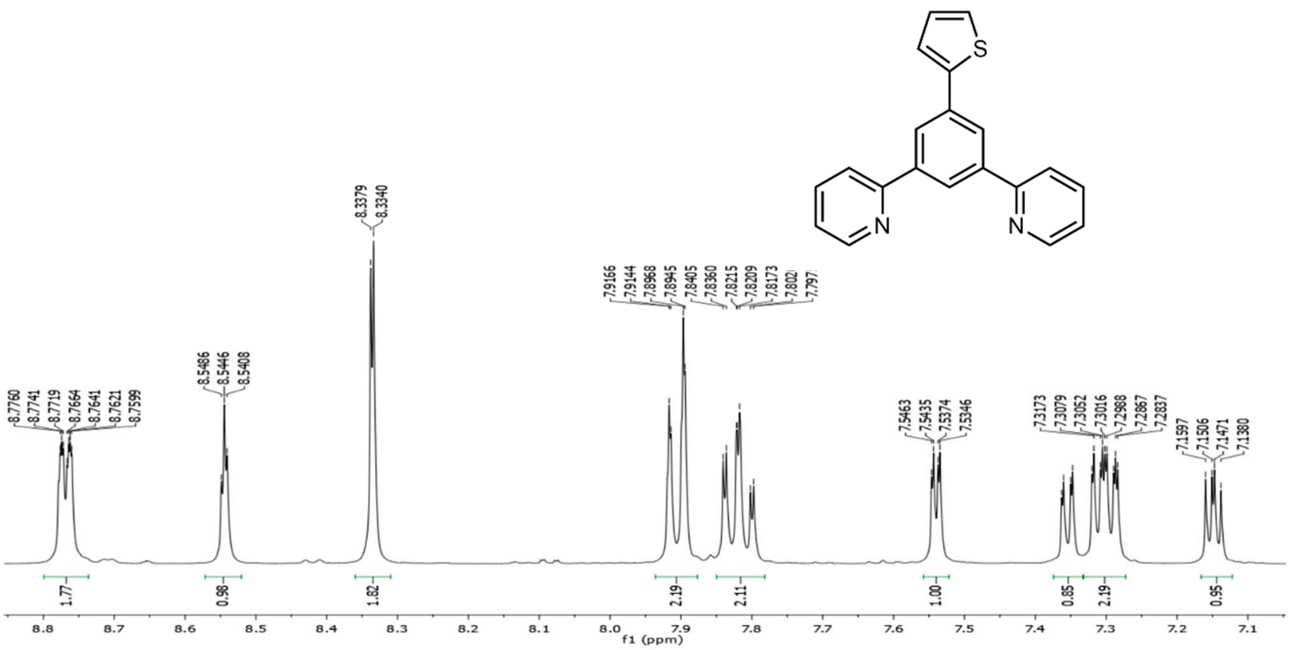

Figure S25. ^{1}H NMR of **L3**.

L4

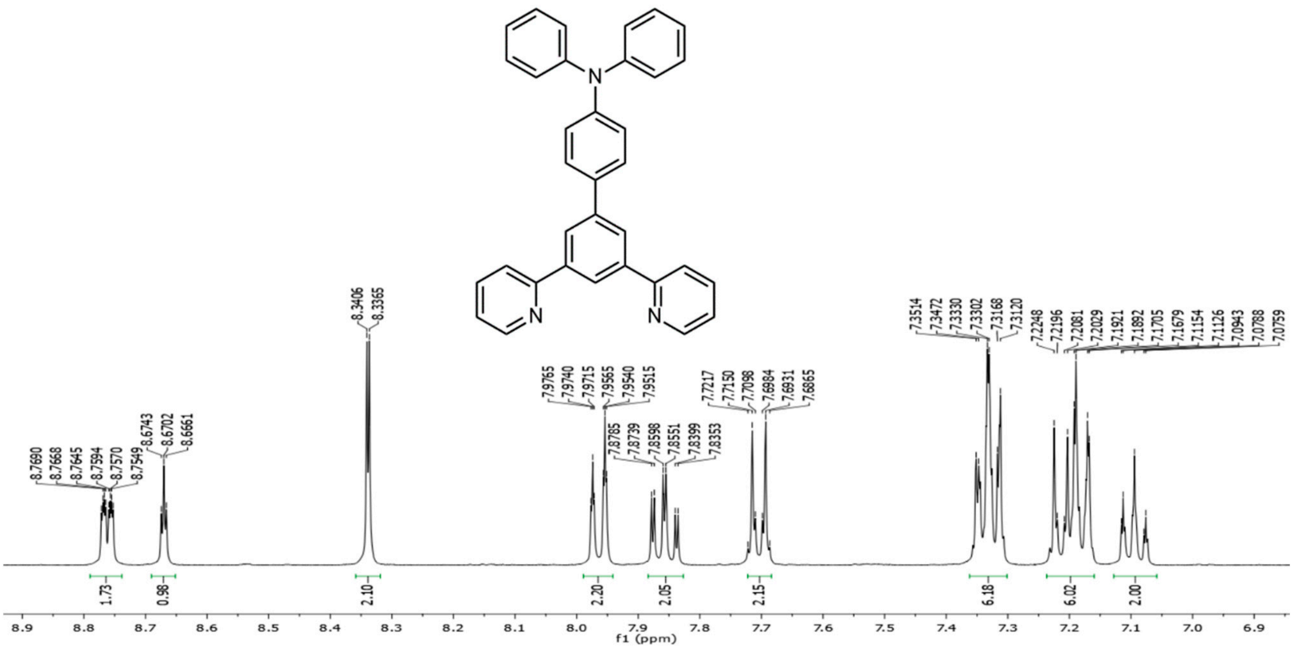

Figure S26. ^{1}H NMR of **L4**.

PtCl1

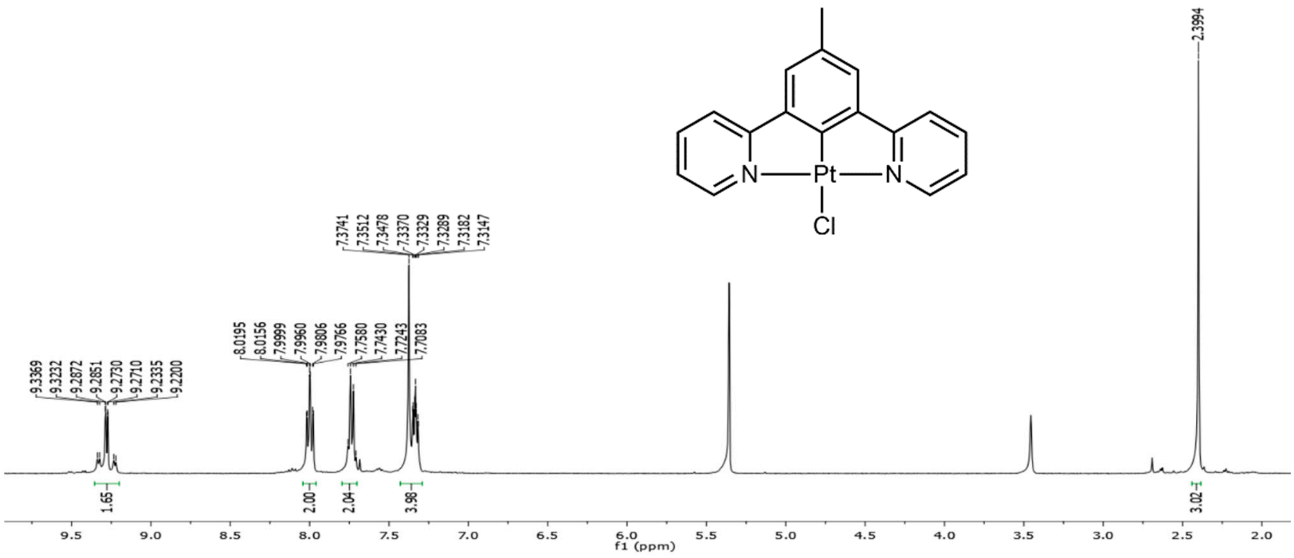

Figure S27. ^{1}H NMR of **PtCl1**.

PtCl1 – aromatic region

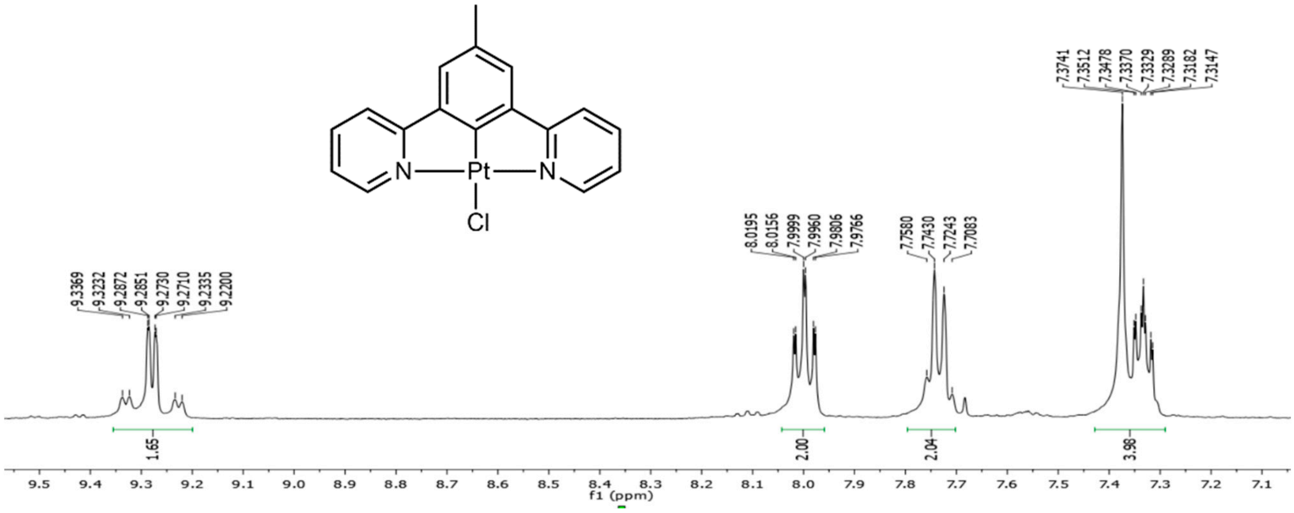

Figure S28. ^{1}H NMR of **PtCl1**, aromatic region.

PtCl2

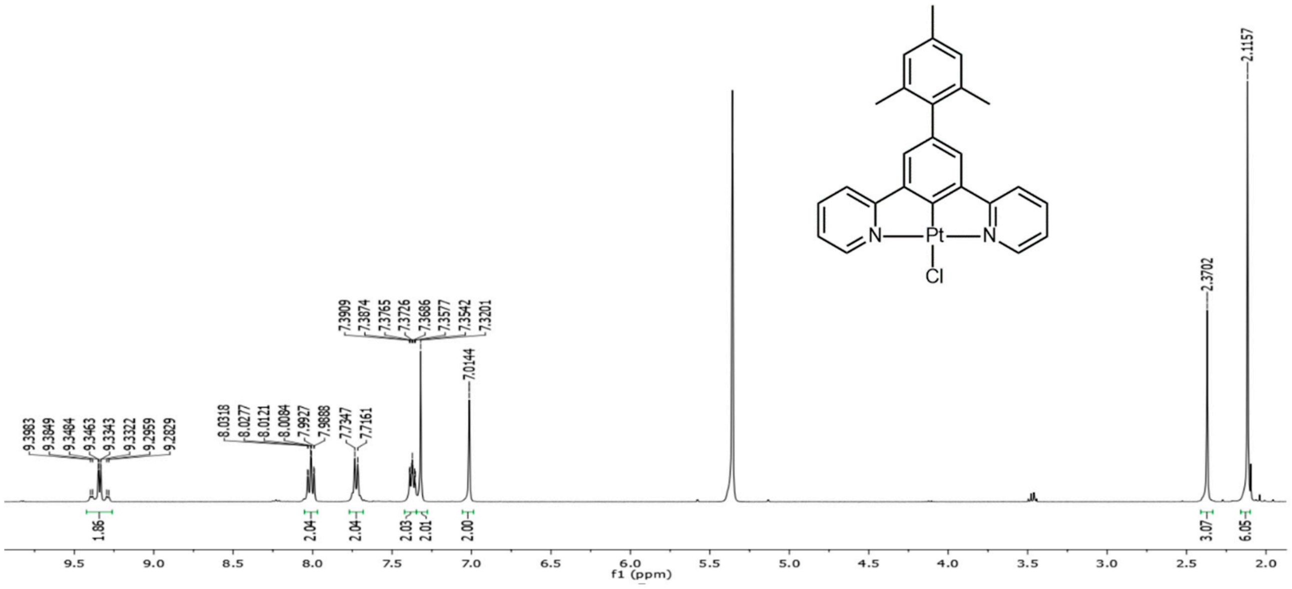

Figure S29. ^{1}H NMR of **PtCl2**.

PtCl2 – aromatic region

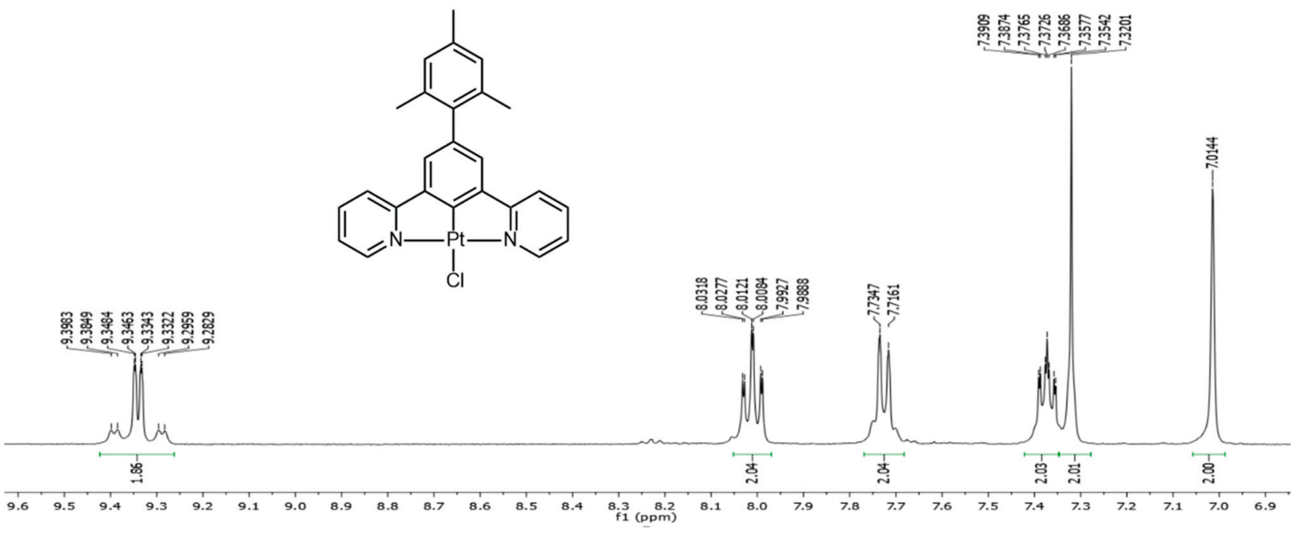

Figure S30. ^{1}H NMR of **PtCl2**, aromatic region.

PtCl3

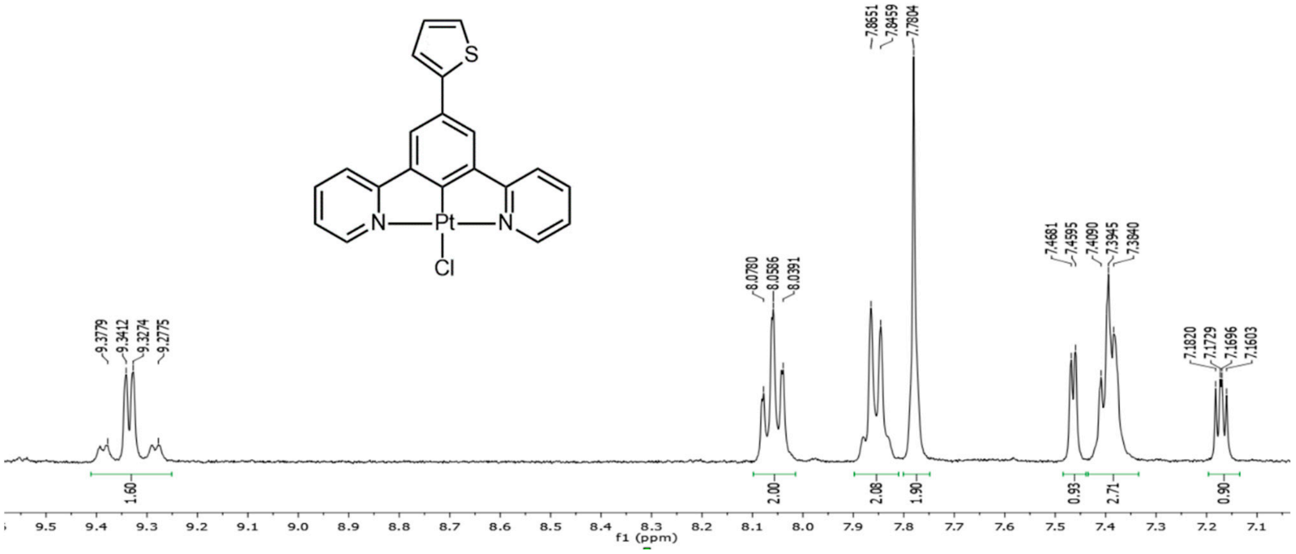

Figure S31. ^{1}H NMR of **PtCl3**.

PtCl4

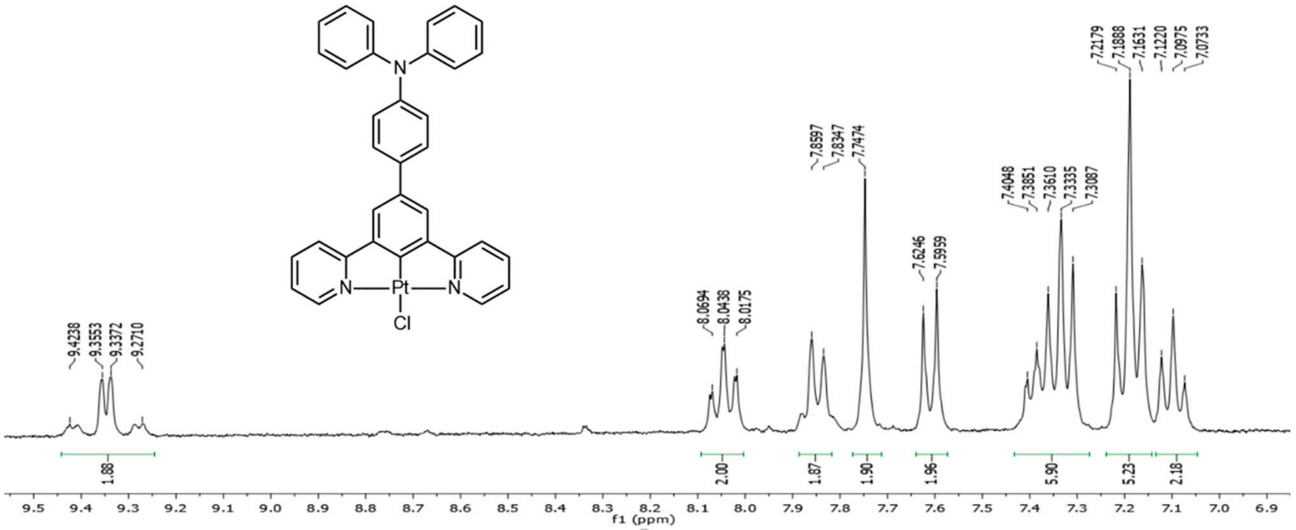

Figure S32. ^{1}H NMR of **PtCl4**.

Pt1

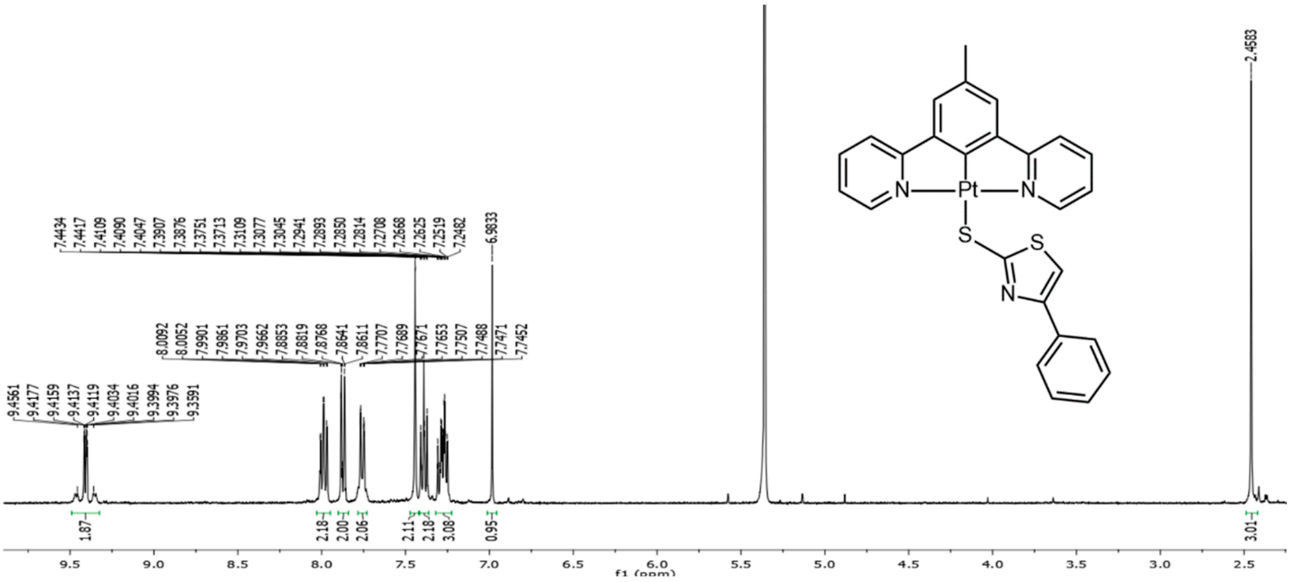

Figure S33. ^{1}H NMR of **Pt1**.

Pt1 – aromatic region

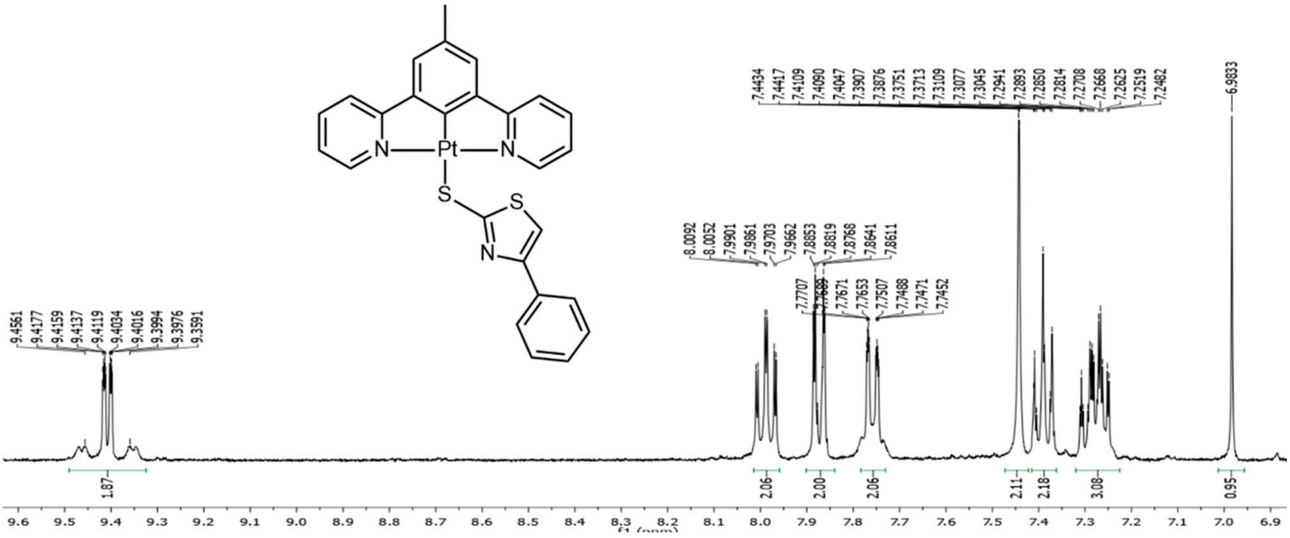

Figure S34. ^{1}H NMR of **Pt1**, aromatic region.

Pt1 – ^{13}C NMR

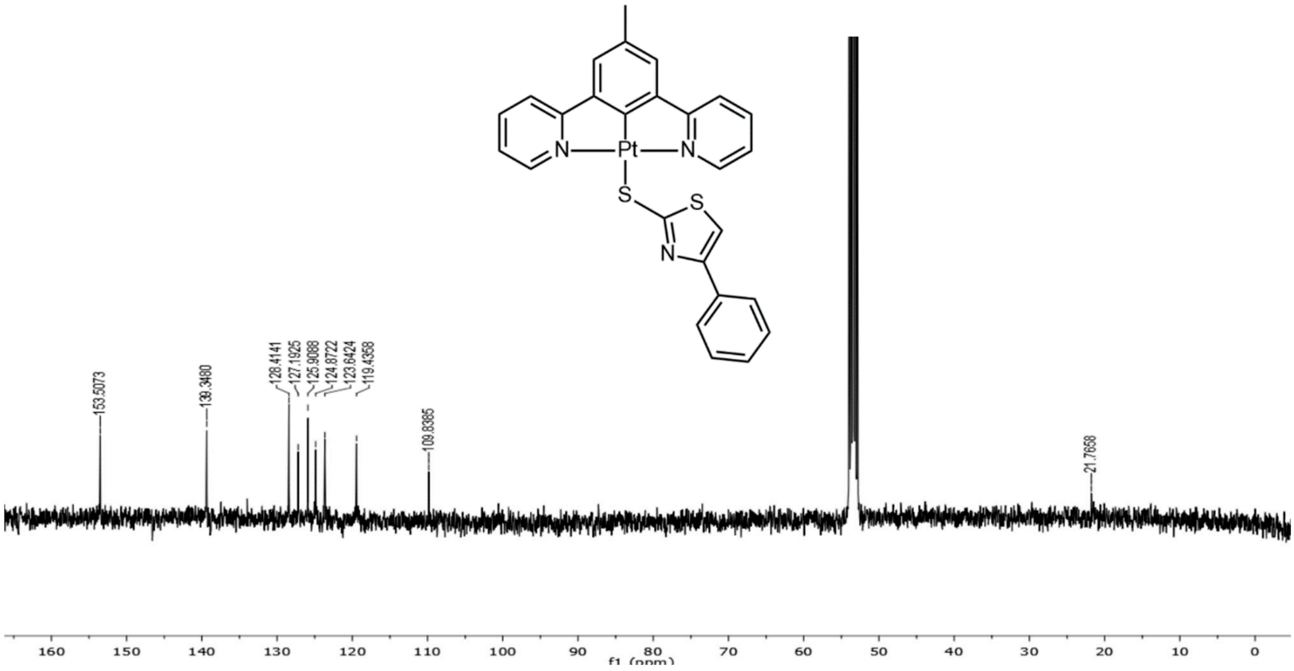

Figure S35. ^{13}C NMR of **Pt1**.

Pt2

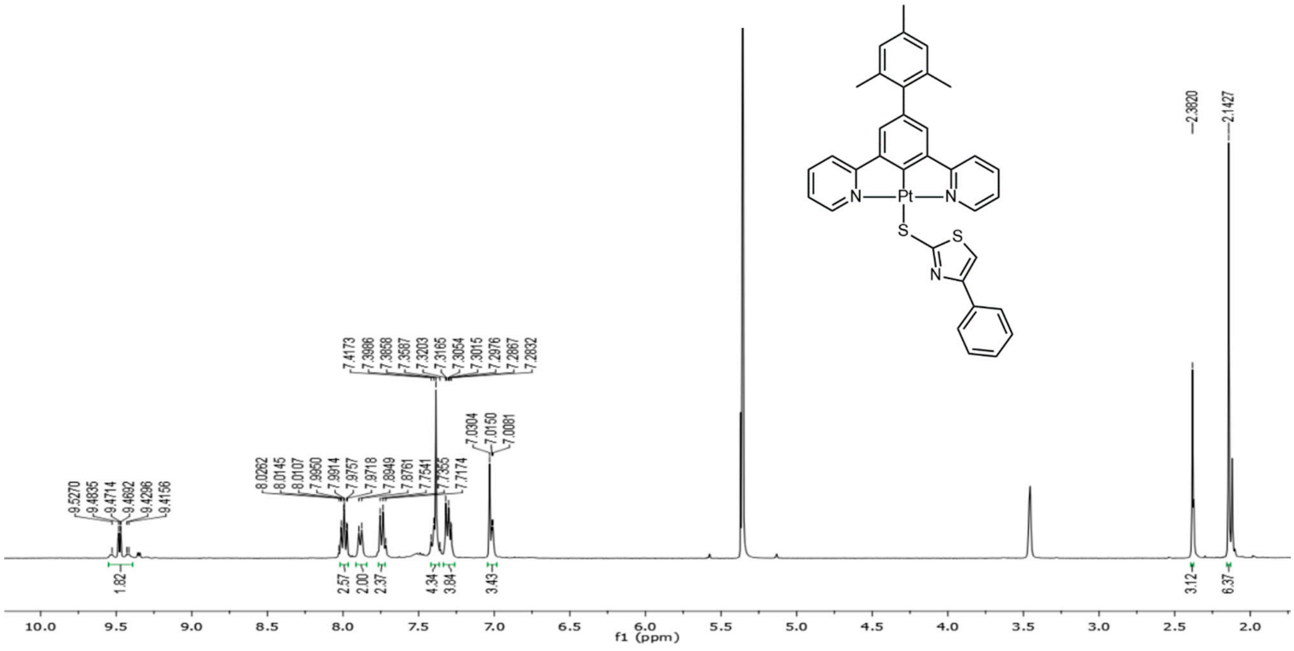

Figure S36. ^{1}H NMR of **Pt2**.

Pt2 – aromatic region

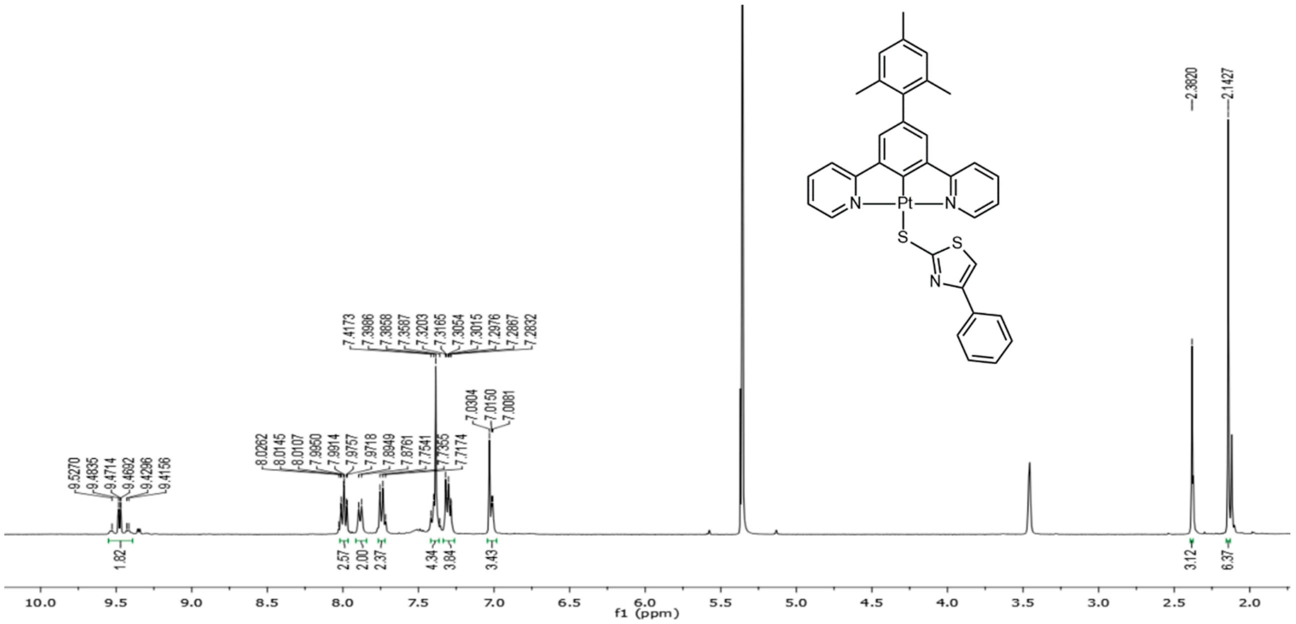

Figure S37. ^{1}H NMR of **Pt2**, aromatic region.

Pt3

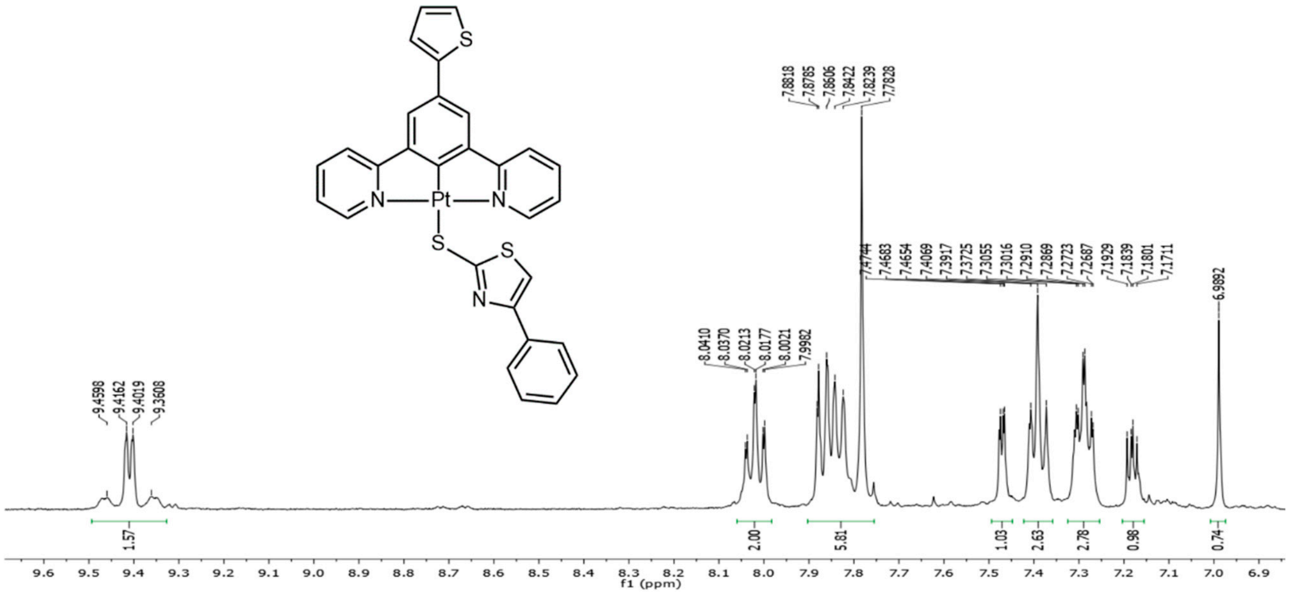

Figure S38. ^{1}H NMR of **Pt3**.

Pt4

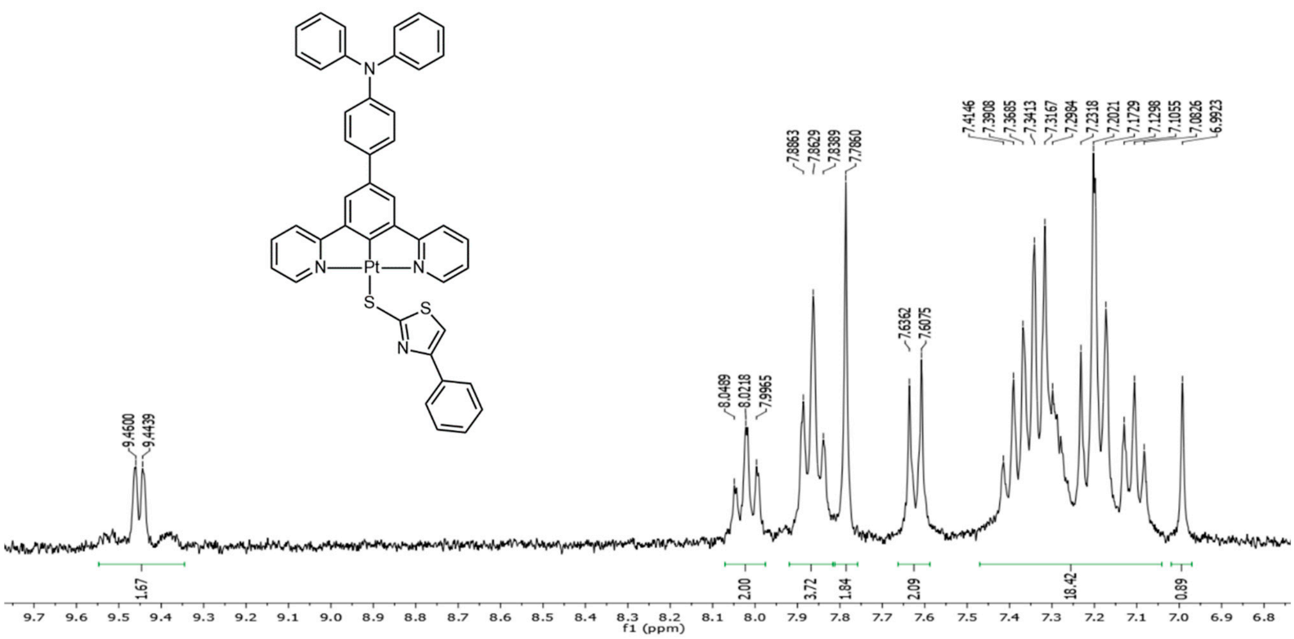

Figure S39. ^{1}H NMR of **Pt4**.

References

1. K. Suzuki, A. Kobayashi, S. Kaneko, K. Takehira, T. Yoshihara, H. Ishida, Y. Shiina, S. Oishic, S. Tobita, "Reevaluation of absolute luminescence quantum yields of standard solutions using a spectrometer with an integrating sphere and a back-thinned CCD detector", *Phys. Chem. Chem. Phys*., **2009**, 11, 9850–9860. DOI: 10.1039/b912178a
